# Fatty acid capped, metal oxo clusters as the smallest conceivable nanocrystal prototypes†

**DOI:** 10.1039/d2sc05037d

**Published:** 2022-12-08

**Authors:** Dietger Van den Eynden, Rohan Pokratath, Jikson Pulparayil Mathew, Eline Goossens, Klaartje De Buysser, Jonathan De Roo

**Affiliations:** a Department of Chemistry, University of Basel Mattenstrasse 24a 4058 Basel Switzerland Jonathan.deroo@unibas.ch; b Department of Chemistry, University of Ghent Krijgslaan 281 9000 Ghent Belgium

## Abstract

Metal oxo clusters of the type M_6_O_4_(OH)_4_(OOCR)_12_ (M = Zr or Hf) are valuable building blocks for materials science. Here, we synthesize a series of zirconium and hafnium oxo clusters with ligands that are typically used to stabilize oxide nanocrystals (fatty acids with long and/or branched chains). The fatty acid capped oxo clusters have a high solubility but do not crystallize, precluding traditional purification and single-crystal XRD analysis. We thus develop alternative purification strategies and we use X-ray total scattering and Pair Distribution Function (PDF) analysis as our main method to elucidate the structure of the cluster core. We identify the correct structure from a series of possible clusters (Zr3, Zr4, Zr6, Zr12, Zr10, and Zr26). Excellent refinements are only obtained when the ligands are part of the structure model. Further evidence for the cluster composition is provided by nuclear magnetic resonance (NMR), infrared spectroscopy (FTIR), thermogravimetry analysis (TGA), and mass spectrometry (MS). We find that hydrogen bonded carboxylic acid is an intrinsic part of the oxo cluster. Using our analytical tools, we elucidate the conversion from a Zr6 monomer to a Zr12 dimer (and *vice versa*), induced by carboxylate ligand exchange. Finally, we compare the catalytic performance of Zr12-oleate clusters with oleate capped, 5.5 nm zirconium oxide nanocrystals in the esterification of oleic acid with ethanol. The oxo clusters present a five times higher reaction rate, due to their higher surface area. Since the oxo clusters are the lower limit of downscaling oxide nanocrystals, we present them as appealing catalytic materials, and as atomically precise model systems. In addition, the lessons learned regarding PDF analysis are applicable to other areas of cluster science as well, from semiconductor and metal clusters, to polyoxometalates.

## Abstract 2

Metal oxo clusters of the type M_6_O_4_(OH)_4_(OOCR)_12_ (M = Zr or Hf) are valuable building blocks for materials science.

## Graphical abstract

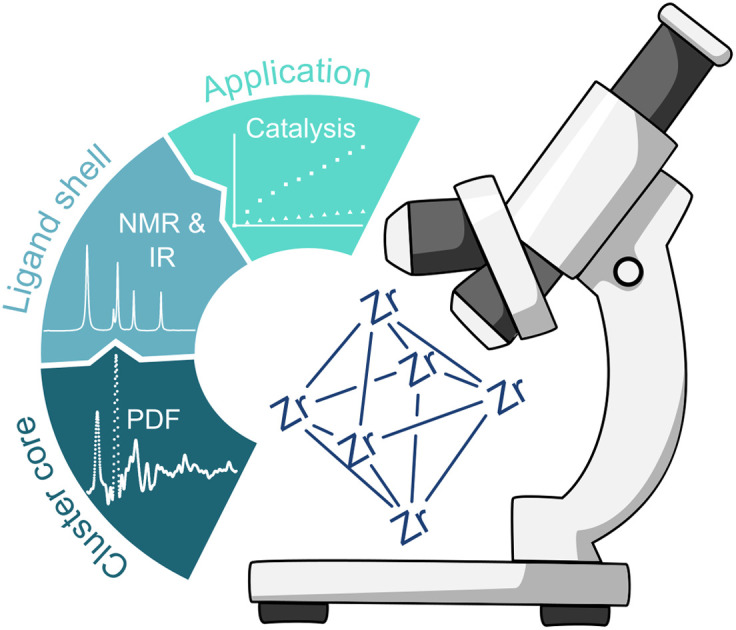

## 1 Introduction

Atomically precise clusters are an exciting material class.^1,2^ They span a broad range from oxo clusters, over metal clusters to semiconducting clusters, *e.g.*, Zr_6_O_4_(OH)_4_(OMc)_12_ (Zr6, OMc = methacrylate),^3^ [Hf_6_O_4_(OH)_4_(OOCMe)_12_]_2_ (Hf12),^4^ Ti_8_O_8_(OOCPh)_16_,^5^ Au_55_(PPh_3_)_12_Cl_6_,^6^ Cd_84_Se_56_(O_2_CPh)_56_(RNH_2_)_56_,^7^ or In_37_P_20_(O_2_CCH_2_Ph)_51_.^8^ Clusters are used to build stimuli-responsive materials,^9^ or hierarchical (porous) structures.^10,11^ Clusters can be precursors or intermediates in nanocrystal synthesis.^12–15^ Composites with improved strength are produced by incorporating zirconium oxo clusters into polymers.^16,17^ Zirconium and hafnium oxo clusters are used as catalysts to make or break amide bonds,^18,19^ and they are suitable as inorganic extreme-ultraviolet photoresists.^20,21^ Subtle differences in cluster chemistry between Zr and Hf are exploited in the industrial separation of Zr from Hf.^22^ Titanium oxo clusters exhibit exciting photo-redox properties.^23^ Group 4 MOFs are based on group 4 metal oxo clusters that act as secondary building units (or nodes) in the MOF framework.^5,24–28^ Apart from the typical Zr6, and Hf6 oxo clusters used in MOFs, many discrete group 4 oxo clusters exist with different nuclearities.^2,29^

**Fig. 1:**
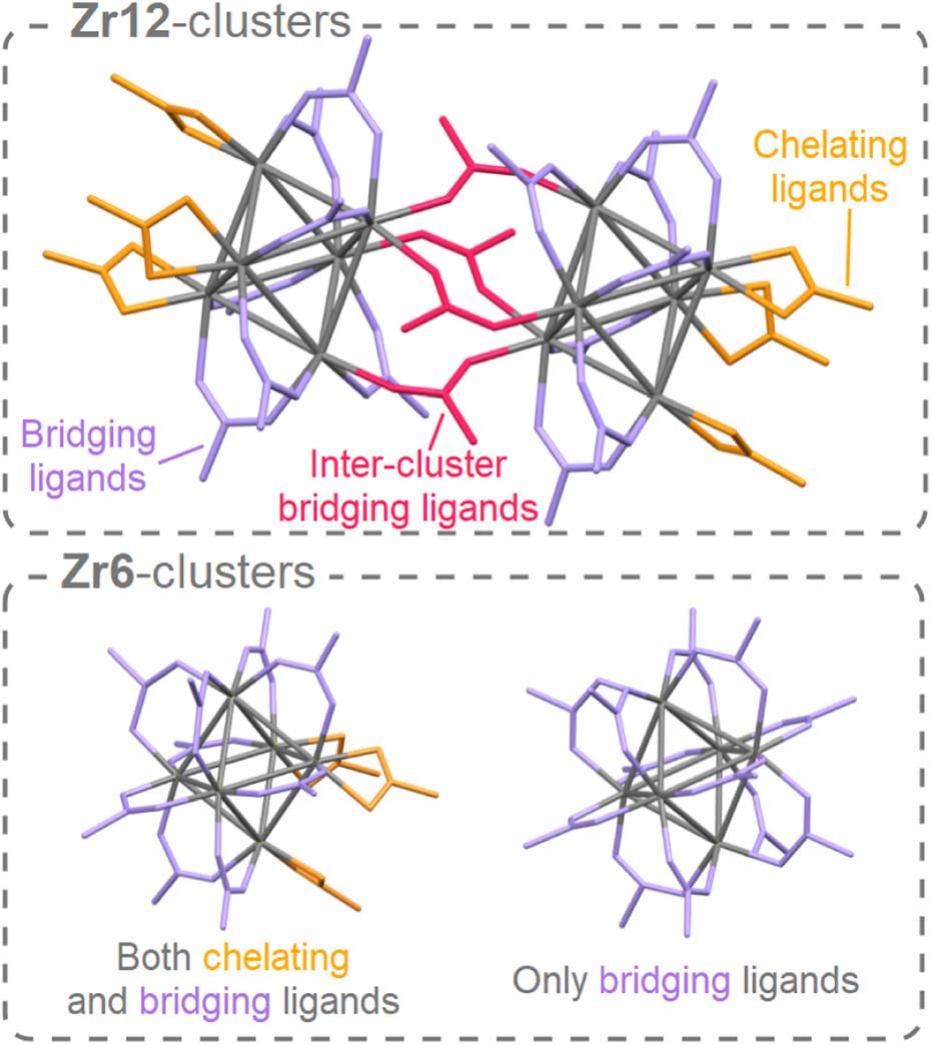
The structure of the Zr12 dimer and Zr6 monomers. For the dimer, there are three different types of ligands: chelating, bridging, and inter-cluster bridging. The latter connects two monomers to form the dimer. A monomer can have either only bridging ligands or a combination of bridging and chelating ligands. For clarity, hydrogen atoms and oxygen atoms on the cluster core are omitted, and ligand chains are shortened to acetate.

Discrete group 4 oxo clusters are conceptually close to colloidal oxide nanocrystals and share some features with them.^2,30,31^ For example, both have an inorganic core, usually capped by an organic ligand shell. Discrete oxo clusters are usually synthesized with short and rigid ligands (*e.g.*, acetate or benzoate). The resulting clusters crystallize from the reaction mixture, allowing for convenient purification and structural characterization by single crystal X-ray diffraction (XRD).^3,32,33^ For example, Zr6-carboxylate clusters have the general formula: Zr_6_O_4_(OH)_4_(OOCR)_12_. The Zr atoms are arranged as an octahedron and are connected through μ_3_-oxygen bridges, with the largest Zr–Zr distance being 0.5 nm. All zirconium atoms are eight-coordinate and the zirconium–oxygen arrangement is almost identical to bulk, cubic zirconia. Therefore, these clusters can be regarded as the lower limit of downscaling zirconia nanocrystals. In contrast to larger nanocrystals, oxo clusters are atomically precise (polydispersity = 0),^34^ and their ligand shell is less densely packed (ligand density = 2.4 ligands per nm^2^ compared to 3–4 ligands per nm^2^ for nanocrystals).^35,36^ When capped by ligands with low steric hindrance, the Zr6 cluster dimerizes to form a Zr12 cluster that has two Zr6 subunits, connected by four inter-cluster bridging carboxylate ligands,^2^ see Fig. 1. Apart from seven regular bridging ligands, there are also three chelating ligands per Zr6 subunit. There exist two types of Zr6 monomer structures, either with all bridging ligands or with three chelating and nine bridging ligands, see Fig. 1. Due to their rigid ligands, typical oxo clusters have low solubility, rendering them poorly suitable for subsequent processing.^37^ Ligands typically used for providing colloidal solubility to nanocrystals (*e.g.*, oleic acid),^38,39^ have not been explored on group 4 oxo clusters. In general, the surface chemistry of group 4 oxo clusters is less studied than that of group 4 oxide nanocrystals.^35,40–44^ Only carboxylate for carboxylate ligand exchange is explored.^4,45^ At room temperature, the Zr6 and Zr12 clusters do not interconvert and the bridging ligands appear inaccessible for exchange.

Drawing inspiration from the nanocrystal field, we envision a library of flexible carboxylate ligands to increase the solubility and hence the possible applications of these clusters. Since longer carboxylate ligands do not allow for crystallization, the question becomes how to purify and structurally characterize such objects. Recently, a purification method based on resins with amine groups was developed for oxo clusters with mixed (but still short) ligand shells.^20^ About 20% of the acid persists as free acid in the final product. Regarding characterization, there are a few examples where oxo clusters in MOFs have been studied by X-ray total scattering and Pair Distribution Function analysis (PDF).^46–49^ Structural changes upon heating have been observed by a model-free analysis. Recently, oxo clusters were modelled *via* PDF analysis without organic ligands in MOF reaction mixtures.^47,49^ On the other hand, X-ray PDF has been often used to study the structure of nanocrystals. In this case, the organic ligands have usually been neglected in the structural models because the organic ligands scatter very little compared to the inorganic core.^50–56^ In the case of larger clusters (35–144 metal atoms) PDF could confirm the structure of the core,^7,57–59^ again by largely neglecting the organic ligands. However, to our knowledge, PDF has not yet been applied to small discrete oxo clusters capped with organic ligands, as discussed here. One can expect this to be challenging since the core is much smaller.

Here, we establish standardized conditions for the synthesis of a range of carboxylate capped zirconium oxo clusters. We use PDF analysis to structurally characterize the oxo clusters. We find that excellent refinements are only obtained when the structural models include the contribution of the organic ligands. PDF analysis can clearly distinguish a Zr6 cluster from a Zr12 cluster and dismiss other possible cluster structures. The organic ligand shell is then comprehensively characterized by FTIR, NMR and TGA. The molecular formula is finally confirmed by electrospray ionization mass spectrometry (ESI-MS). Using our characterization tools, we evaluate carboxylate for carboxylate ligand exchange and we find that the ligand is structure-directing, determining whether a Zr6 or a Zr12 cluster is formed. These results are further generalized to hafnium oxo clusters. Finally, we test the notion that the fatty acid capped metal oxo clusters are the smallest conceivable nanocrystal prototypes. We compare the catalytic activity of Zr12-oleate clusters with oleic acid capped zirconium oxide nanocrystals, and find a superior activity for the oxo clusters.

## 2 Results and discussion

### 2.1 Synthesis of oxo clusters

**Fig. 2:**
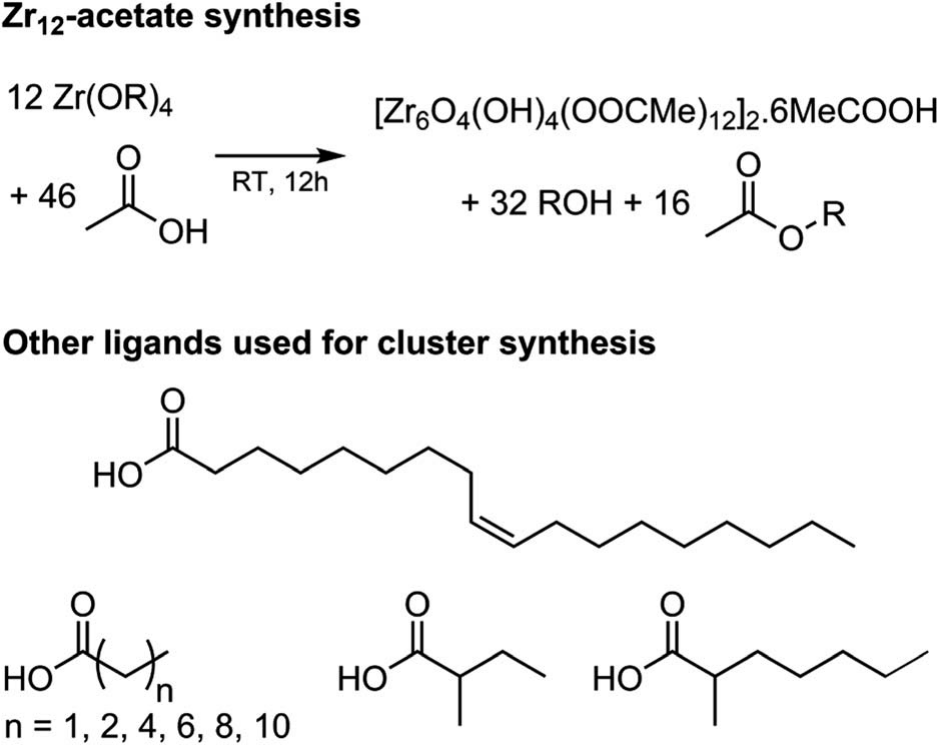
Balanced chemical equation for the synthesis of Zr12-acetate clusters from zirconium propoxide (or butoxide) and acetic acid. The final product has co-crystallized acetic acid and dichloromethane (the latter is not shown). Also the structures of the other carboxylic acids used here for cluster synthesis are shown.

Zirconium propoxide (70 w% in propanol) or zirconium butoxide (80 w% in butanol) reacts with an excess of acetic acid (8 equivalents) to form a Zr12-acetate cluster, which is a dimer of Zr6.^4^ The reaction is performed in dichloromethane (DCM) solvent to obtain higher quality crystals.^4^ Both acetic acid and DCM are co-crystallized with the cluster.^4^ The balanced chemical equation is shown in Fig. 2. We further washed the crystalline powder with a solution of acetic acid in DCM to remove reaction by-products and other zirconium species. Although the powder can be handled in air, it is recommended to keep the clusters in a glovebox or desiccator for long term storage, since they react slowly with atmospheric water and become partially insoluble.

Zr12-propionate is similarly synthesized and purified as Zr12-acetate. We extended the synthetic strategy to longer carboxylic acid ligands (see Fig. 2), but the resulting clusters cannot be crystallized anymore and therefore other purification techniques are required. Zr12-butanoate is washed overnight in acetonitrile, while Zr12-hexanoate, Zr6-methylbutyrate and Zr6-methylheptanoate clusters can be precipitated with acetonitrile and isolated by centrifugation. Dissolution in DCM and repeating the precipitation cycle twice, delivers the purified clusters. This type of purification is typical for colloidal nanocrystals,^60^ and thus reinforces the idea of fatty acid capped clusters as atomically precise models for nanocrystals. For the clusters with even longer ligands (octyl, decyl, dodecyl and oleyl chains), the first precipitation is performed with acetonitrile, while acetone is the preferred anti-solvent for the two consecutive steps to precipitate the clusters, and separate it from, *e.g.*, the ester co-product. The physical appearance of the clusters ranges from semi-crystalline solids (acetate, propionate and methylbutanoate) to waxy solids (butanoate, hexanoate, decanoate and dodecanoate) or viscous oils (octanoate, oleate and methylheptanoate), see Fig. S1.†

### 2.2 Structural analysis by X-ray PDF

**Fig. 3:**
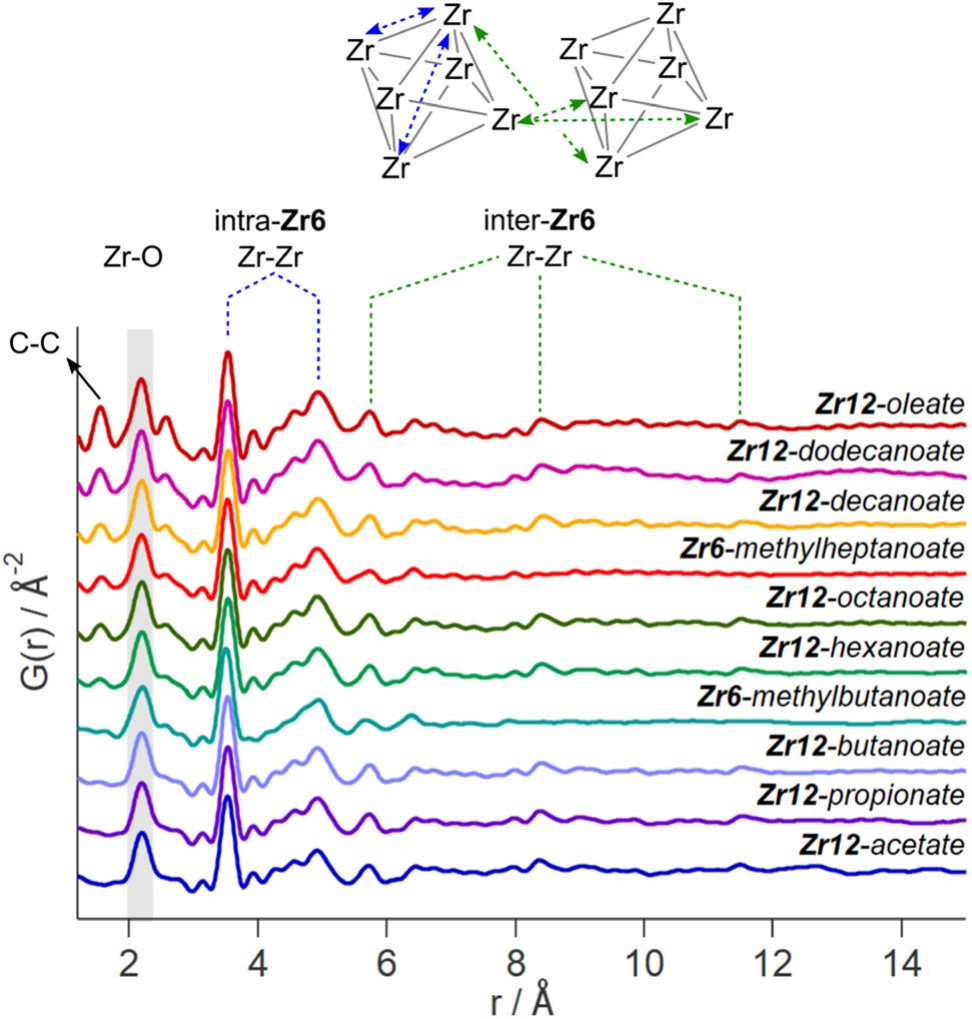
Experimental PDF of zirconium oxo clusters with different capping ligands. The C–C, Zr–O, and Zr–Zr distances are assigned. We make a distinction between the Zr–Zr distances within one Zr6 cluster and the Zr–Zr distances that are characteristic for the dimer.

While the Zr12-acetate and Zr12-propionate clusters are known in the literature, the others are not. To prove their structure, we analyzed the clusters with X-ray total scattering and Pair Distribution Function (PDF) analysis. Total scattering takes into account both Bragg and diffuse scattering. The real space PDF is ideal to study nanomaterials, local effects and amorphous materials.^61–63^ We measured the experimental PDF of all the above clusters and ordered them according to increasing carbon contents, see Fig. 3. Focusing first on the PDF of acetate capped clusters and comparing it with the structure from single crystal XRD,^4^ we easily recognize the Zr–O distance at 2.2 Å and the Zr–Zr distances within one Zr6 cluster at 3.5 Å and 5 Å. We label them as intra-Zr6 distances. The longer range Zr–Zr distances are specific to the Zr12 dimer and are present at 5.7 Å, 8.4 Å, and 11.5 Å. They are labeled as inter-Zr6 distances. When the chain length increases, the same features remain present in the PDF, except for the case of methylbutanoate and methylheptanoate where the inter-Zr6 distances are missing. The peak at 5.7 Å does not completely disappear but changes shape and has a lower intensity. The residual intensity stems from intra-Zr6 Zr–O distances (not the first coordination sphere but longer range correlations). The above observations suggest that both the Zr6-methylbutanoate and Zr6-methylheptanoate clusters are monomers while the others are dimers. This is in agreement with our earlier hypothesis that branching of the carboxylic acid at the alpha position is the deciding factor between the monomer and dimer.^2^ On analyzing the theoretical structure of Zr12-isobutyrate (SI), the steric hindrance is clear from the space filling representation. On the other hand, a higher degree of oligomerization was excluded based on the absence of features beyond 11.5 Å, and based on the crystal structure of Zr12-acetate. With increasing chain lengths, we also observe the growth of a peak at 1.5 Å, which we assign to C–C distances in the ligand chain.

**Fig. 4:**
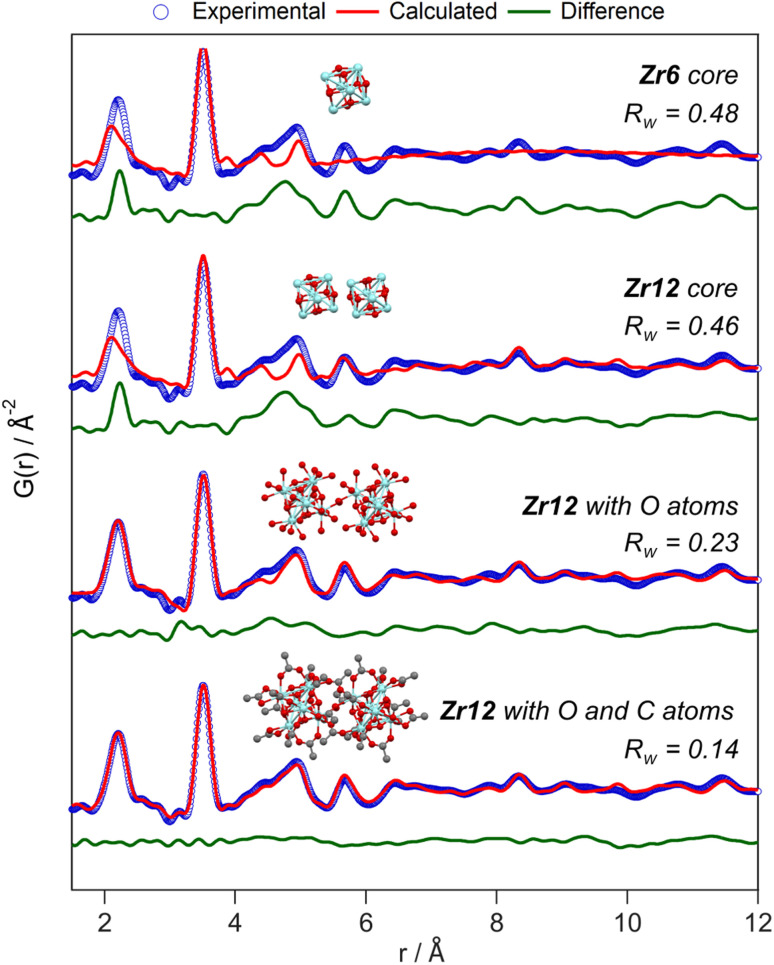
PDF refinement of the Zr12-acetate cluster with various models, derived from the reported crystal structure.^4^ The best fit is obtained when including the oxygen and carbon atoms from the acetate ligands. The refined parameters are given in Table S1.†

Apart from this model-free analysis, we sought to quantitatively fit the PDFs. We focus again first on Zr12-acetate to refine our approach since a single crystal XRD structure is available for this cluster (CCDC-604528).^4^ To demonstrate the importance of the organic ligands to the PDF, we theoretically calculated the PDF of Zr12-acetate, including either (i) no carbon atoms, (ii) only the carbonyl carbon atoms, or (iii) all carbon atoms, see Fig. S2.† The carbon atoms contribute significantly to the PDF, especially between 4 Å and 8 Å. This result stands in stark contrast to the regular practice of ignoring the ligands in the PDF refinement of nanocrystals and larger clusters.^7,50–59^ The ligand effect is further demonstrated by fitting the Zr12-acetate PDF with various models, see Fig. 4. For each structure model, we refined thermal motion parameters while keeping the structure (*i.e.*, the atomic positions) unchanged. In the first attempt, similar to traditional approaches, we only used the Zr6 cluster core as a model. The model could capture the basic Zr–O and intra-Zr6 Zr–Zr distances but gave overall a poor agreement and a significant misfit in the Zr–O distance. Using the dimer structure, the inter-Zr6 Zr–Zr distances are well described. By including the oxygen atoms from coordinated acetates, a significant reduction in *R*_w_ is achieved and the Zr–O peak is now accurately fitted. By including the carbon atoms from coordinated acetate, we obtain a decent fit, with *R*_w_ = 0.14.

The crystal structure of Zr12-acetate contains also hydrogen bonded ligands and co-crystallized DCM molecules. When the hydrogen bonded ligands are explicitly included, only a marginally better fit is obtained, see Fig. S3.† Alternatively, we added an exponentially decaying sinusoidal function as a second phase, resulting in a slightly (but significantly) better fit with an *R*_w_ value of 0.11, see Fig. S4.† This second phase describes the disordered but still structured intensity and has previously been used to model solvent restructuring, or a regular array of high/low density regions.^64,65^ While it is gratifying to obtain an excellent fit for the data, we sought to determine whether we could distinguish the Zr12-acetate structure from other reported cluster structures (determined by single crystal XRD). We thus attempted to fit the experimental PDF of Zr12-acetate clusters with the reported structures of Zr12-propionate, Zr6-trimethylacetate, Zr6-methacrylate, Zr6-isobutyrate, Zr6-acetate, Zr4-methacrylate, Zr4-formate isopropoxide, Zr3-acetate isopropoxide, Zr3-acetate *tert*-butoxide, Zr10-salicylate, and Zr26-formate, see Fig. S5.†^3,4,32,66–70^ For these refinements, we removed the excess carbons in the structures to arrive at models with acetate ligands. The models based on Zr3, Zr4, Zr10 and Zr26 all showed very poor agreement with the data. The Zr6 structure models also delivered poor fits (*R*_w_ = 0.24–0.31). The second best agreement was obtained for the Zr12-propionate model (*R*_w_ = 0.18). While this model is structurally very similar to the Zr12-acetate model, the Zr–Zr distances are slightly larger in the propionate model (Table S6†). We conclude that PDF is able to pick up the correct structure from a series of possible cluster structures, based on the goodness of fit.

**Fig. 5:**
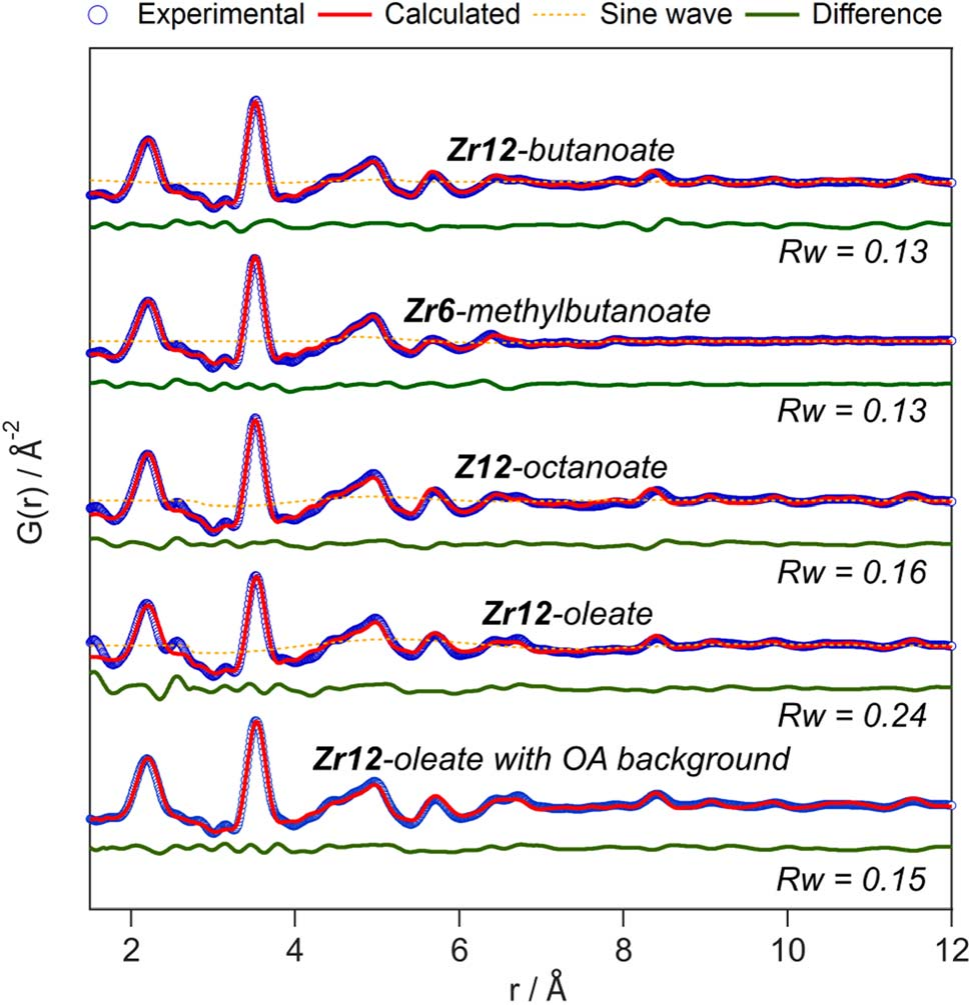
PDF refinement for Zr12-butanoate and Zr12-octanoate using the Zr12-acetate structure model. PDF refinement of Zr6-methylbutanoate using the Zr6-acetate structure model. Finally, the PDF refinement of Zr12-oleate using the Zr12-propionate structure model, with or without background correction in reciprocal space. If applied in the refinement, the exponentially dampening sine wave is shown (orange dotted lines). The refined parameters are given in Table S3.†

The developed strategy is now applied to the other oxo clusters. Fig. 5 shows the PDF refinement for four selected oxo clusters, capped with either butanoate, methylbutanoate, octanoate or oleate. In each case, we use a cluster model along with a decaying sine wave. For Zr12-butanoate and Zr12-octanoate, we find that the Zr12-acetate structure model gives a satisfactory fit (Fig. 5), which is clearly better than the refinement with a Zr6-acetate structural model (Fig. S6†). The latter structure has all bridging ligands and was reported after crystallization from an aqueous solution.^66^ In contrast, the Zr6-methylbutanoate cluster, is best described by using the Zr6 model and gives a worse fit for the Zr12 model. The Zr12-oleate cluster was best described by using the Zr12-propionate structure model with slightly larger Zr–Zr distances. Again, the Zr6 model delivered a worse fit (Fig. S6†). We can thus distinguish the Zr6 structures from the Zr12 structures, based on the refinement. However, we notice that the *R*_w_ increases with increasing chain length (indicating a worse fit). The origin lies in the fact that we did not explicitly include all the carbons of the long ligands in the models. The short-range C–C distances (at 1.5 and 2.6 Å) are not fitted in the refinement. However, the sinusoidal function seems to describe the long range C–C distances quite well, presumably because the ligand chain is disordered. This hypothesis is confirmed by the increase in the relative amplitude of the sine wave from Zr12-acetate to Zr12-oleate (Tables S1 and S3†). The improvement in *R*_w_ upon including the sinusoid is small for acetate (0.03) and large for oleate (0.09) (see Fig. S7†).

A more effective way of dealing with long ligands is measuring the free ligand explicitly as a background and subtracting its scattering from the data in reciprocal space. For Zr12-oleate, this leads to a significant improvement in the fit. The short range C–C distances disappeared from the PDF (Fig. 5) and the sine wave is completely obsolete (Table S3†). The goodness of fit is now comparable to that of the case of the clusters with shorter chains. Note that the presence of long ligands reduces the signal-to-noise ratio during the data collection (due to a lower fraction of oxo clusters in the X-ray beam). We conclude that, after initial training on the acetate clusters, our PDF analysis is able to confirm the structure of these previously unreported clusters. Our synthetic procedures indeed lead to the formation of soluble Zr6 oxo clusters, forming monomers or dimers depending on the steric hindrance of the ligand. We further emphasize that good refinements can only be achieved by including the binding group of the ligand explicitly in the model (even after background subtraction). This is much more important for the small clusters discussed here, than for larger clusters or nanocrystals. For the latter two, there is a larger core of strongly scattering atoms, and relatively fewer, weakly scattering ligands. However, small nanocrystals have typically worse a goodness of fit compared to larger ones and it might be worth considering including ligands explicitly to obtain better refinements.^50^

### 2.3 The organic ligand shell

**Fig. 6:**
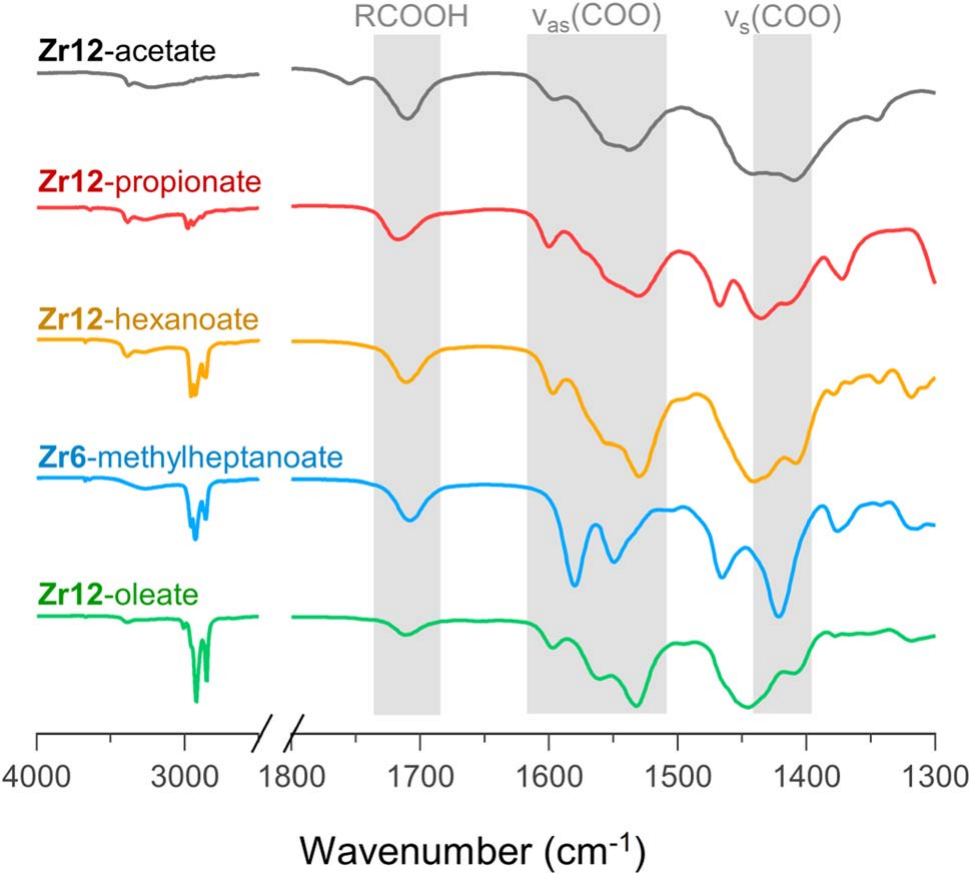
FTIR spectra of Zr12-acetate, -propionate, -hexanoate, and -oleate and Zr6-methylheptanoate. The weak band at 1750 cm^−1^ in the spectrum of Zr12-acetate is assigned to a small amount of acetic acid that is not involved in hydrogen bonding of any kind.^71^ An ester impurity is ruled out because of the absence of any signal at around 4 ppm in NMR, (see Fig. S10†).

For PDF modelling we need to include the carboxylate head group, but PDF is in general not very sensitive to the organic fraction. Detailed information on the presence of uncoordinated ligands or coordination modes is hard to obtain from PDF. We turn now to FTIR, TGA and NMR. In Fig. 6, we show the FTIR spectra of selected oxo clusters (the complete FTIR spectra of all clusters are presented in Fig. S8†). The CH_2_ stretches (2800–3000 cm^−1^) gain intensity with increasing chain length. The small, sharp peak at around 3450 cm^−1^ and the broad peak between 3500 and 3000 cm^−1^ were assigned to the μ_3_-OH moieties on the cluster core, which are involved in hydrogen bonding. While all these samples were extensively purified by multiple precipitation/re-dissolution cycles, it is clear that there is still protonated carboxylic acid in the sample (observed at 1710 cm^−1^), presumably hydrogen bonded to the cluster. Such hydrogen bonding is also observed for the co-crystallized acetic acid in the single-crystal structure of Zr12-acetate, see Fig. S9.† Our efforts to purify the clusters further and remove the hydrogen bonded acid were not successful and even compromised the integrity of the cluster core, see the ESI.† Since attenuated total reflection (ATR) FTIR is not quantitative, we used TGA to quantify the amount of excess carboxylic acid. We find about 1.1–3.7 excess ligand molecules per Zr6 monomer, see the ESI.† There is no clear trend among the different clusters. Only Zr12-acetate stands out with 5.5 excess ligands per monomer.

Returning to the FTIR spectrum, the bands in the region 1500–1600 cm^−1^ are assigned to the asymmetric stretch of the carboxylate, *ν*_as_(COO). While the symmetric stretch, *ν*_s_(COO), is expected at around 1400–1450 cm^−1^, there is significant overlap of CH_2_ and CH_3_ deformation bands which are expected at around 1440–1460. Focusing here on the qualitative differences, we first draw attention to the spectrum of Zr6-methylheptanoate. In contrast to the IR spectrum of Zr6-pivalate,^72^*ν*_as_(COO) is split into two bands (1580 and 1550 cm^−1^).While Zr6-pivalate has all bridging carboxylates, the band splitting for Zr6-methylheptanoate indicates that part of the methylheptanoate ligands are in the chelating mode. When comparing the pattern of the *ν*_as_(COO) bands in Zr6-methylheptanoate with those of Zr12-oleate, we find striking differences. For Zr6-methylheptanoate, the most intense band is at 1580 cm^−1^ while for Zr12-oleate, the most intense band is at 1532 cm^−1^. In addition, Zr12-oleate has a band with the highest wavenumber, 1597 cm^−1^. Typically the wavelength difference between *ν*_as_(COO) and *ν*_s_(COO) is used as a diagnostic tool to distinguish between chelating (Δ*ν* < 110 cm^−1^), bridging (Δ*ν* = 140–190 cm^−1^) or mondentate (Δ*ν* = 200–320 cm^−1^) binding modes.^73,74^ This is an empirical rule-of-thumb and in the case of multiple bands it is difficult to assign the paired *ν*_s_(COO). With these limitations in mind, we tentatively calculate Δ*ν* = 190 cm^−1^ for the band at 1597 cm^−1^, and assign it to the ligands that bridge the two clusters. More importantly, the same *ν*_as_(COO) pattern of Zr12-oleate is retrieved for all the Zr12 clusters (see also Fig. S8†). Furthermore, the *ν*_as_(COO) pattern of Zr6-methylheptanoate is quasi identical to that of Zr6-methylbutanoate. This is an exciting observation since it provides a very fast method to distinguish between the Zr6 and Zr12 clusters.

**Fig. 7:**
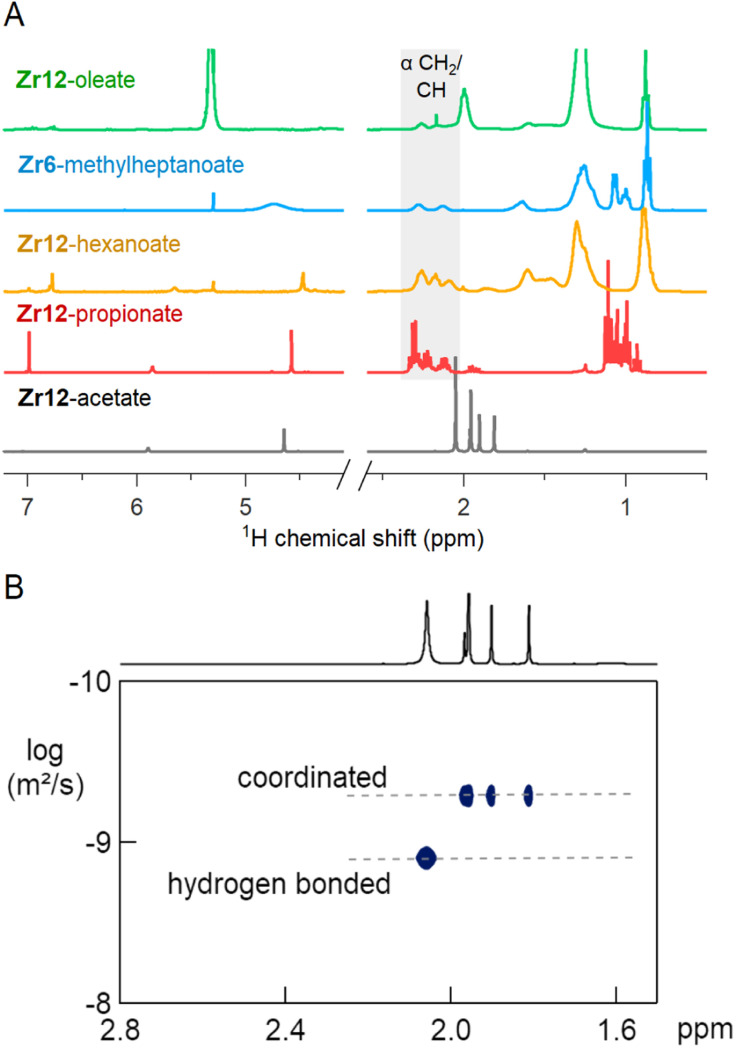
(A) NMR spectra in CDCl_3_ of Zr12-acetate, -propionate, -hexanoate and -oleate and Zr6-methylheptanoate. (B) DOSY of the Zr12-acetate cluster with one faster diffusing species and a set of three resonances pertaining to a slowly diffusing species (the cluster).

The ^1^H NMR spectra of the selected oxo clusters are shown in Fig. 7A. For Zr12-propionate, hexanoate and oleate, we recognize three different resonances for the alpha CH_2_ moiety, indicating three different distinguishable carboxylate environments (see also Fig. S13† for the other Zr12 clusters). On the other hand, Zr6-methylheptanoate has only two alpha CH resonances (see Fig. S13† for Zr6-methylbutanoate). It is striking that the NMR pattern seems to roughly agree with the *ν*_as_(COO) pattern in FTIR. For Zr12-acetate, we find four methyl resonances. Diffusion ordered spectroscopy (DOSY), shows that three resonances (1.7–2 ppm) have the same diffusion coefficient, while the fourth (2.1 ppm) diffuses more quickly, see Fig. 7B. The resonance at 2.1 ppm thus corresponds to excess acetic acid, as also determined by FTIR and TGA. The measured diffusion coefficient (*D* = 1106 μm s^−1^) is lower than the reference of free acetic acid (*D* = 1851 μm s^−1^), and we conclude that the excess acetic acid is in fast exchange between two states: (i) free and (ii) hydrogen bonded to the cluster.^75^ The signals between 5 and 7 ppm in all cluster spectra are assigned to the exchangeable protons on the cluster core itself since the resonances disappear upon addition of D_2_O, see Fig. S14.† Apart from Zr12-acetate and -propionate, all the spectra show significant line broadening, similar to nanocrystal-bound ligands.^76^ The longer the ligands, the broader the alpha CH_2_ resonance, and the sharper the methyl resonance becomes. Homogeneous line broadening depends on the rotational mobility and it is expected that the methyl group has increased mobility in a longer ligand.^76^ The alpha resonance is closest to the surface. Its mobility is not enhanced in a longer ligand and instead, the total rotational mobility of the cluster decreases since it becomes a bigger object. In contrast to nanocrystal-bound ligands, we find little contribution of heterogeneous broadening to the resonances, see Table S8.†

### 2.4 The molecular formula by mass spectrometry

**Fig. 8:**
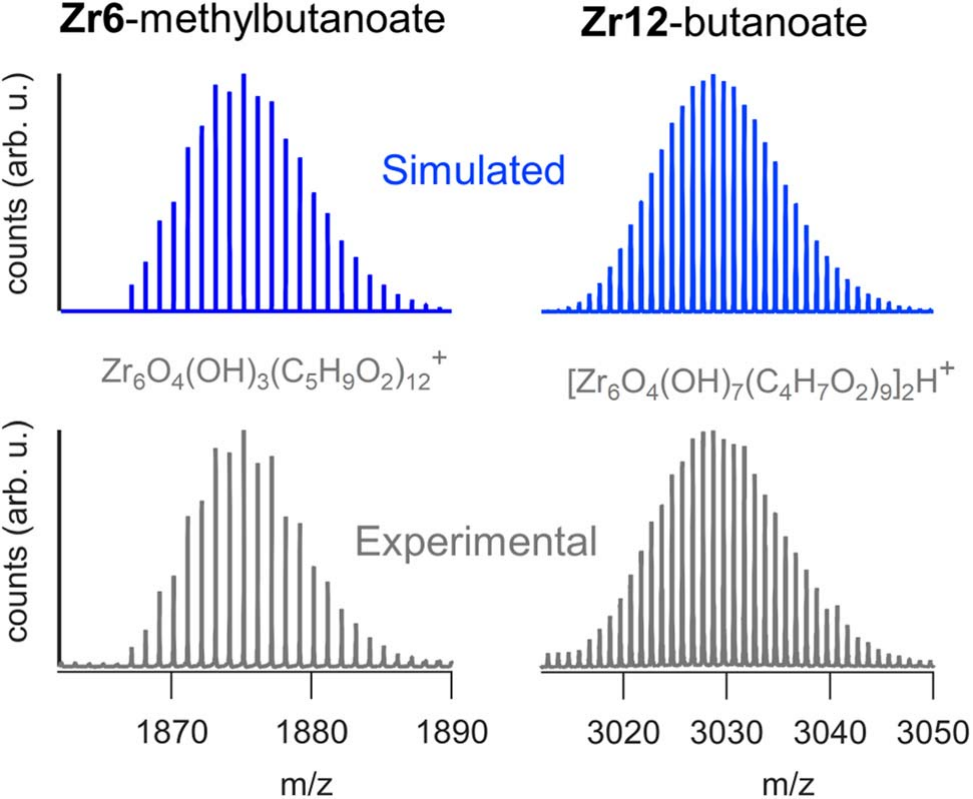
ESI-HR-MS analysis of the dimeric Zr12-butanoate cluster and of the monomeric Zr6-methylbutanoate cluster. Both the experimental and simulated spectra are shown.

We sought to use electrospray ionization high resolution mass spectrometry (ESI-HR-MS) to obtain the mass of the clusters. Unfortunately, our carboxylate capped clusters show limited solubility in typical ESI-HR-MS solvents such as methanol and acetonitrile. Zr12-acetate clusters dissolve in methanol but no clusters were detected in ESI-HR-MS and NMR analysis provides evidence for cluster degradation in methanol, see Fig. S16 and S17.† The limited solubility of Zr12-acetate did not allow for any further HR-MS analysis. On the other end, the Zr12-oleate clusters were too non-polar. The other clusters could be dissolved in tetrahydrofuran (THF) and were analyzed with ESI-HR-MS after adding acetonitrile as co-solvent (full spectra and assignments in Fig. S18–S25†). The Zr6 clusters give reasonably clean spectra where the molecular ion can be recognized as Zr_6_O_4_(OH)_3_(L)_12_+ (the cluster minus an OH^−^ group). The example of Zr6-methylbutanoate is shown in Fig. 8. The Zr12 clusters appear less stable and the ion [Zr_6_O_4_(OH)_7_(L)_9_]_2_H^+^ was observed in most cases (see Fig. 8). This ion is the proton adduct of the Zr12 dimer after substituting six carboxylates for six hydroxides. The Zr12 cluster indeed features 6 chelating ligands, which are most labile according to the literature.^4^ It is thus plausible that such an exchange takes place, further catalyzed by a coordinating solvent like THF. Interestingly, more fragmentation was seen for longer ligands and the monomeric form is even dominant for Zr12-decanoate and Zr12-dodecanoate. Despite the limitations, MS thus confirms the successful formation of oxo clusters with long carboxylate ligands. It is however not the correct technique to distinguish the dimer from the monomer structure.

### 2.5 Ligand exchange

**Fig. 9:**
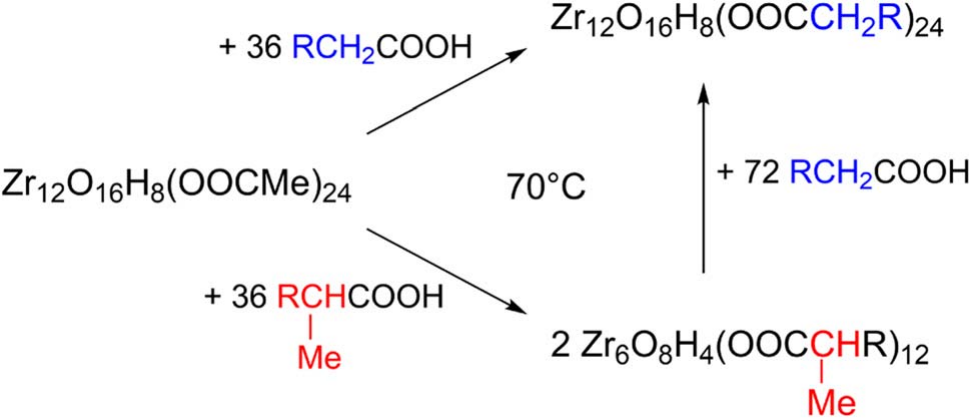
Exchange reaction of the Zr12-acetate cluster with hexanoic acid, and methylbutanoic acid.

Ligand exchange where all carboxylates are replaced by another type of carboxylate is an interesting way to change the cluster structure and/or functionality. Pucherberger *et al.* previously studied the exchange between methacrylate and propionate/acetate on zirconium oxo clusters by NMR. They inferred that, at room temperature, the monomer and dimer cannot be converted into one another and that the inter-cluster bridging ligands are unavailable for exchange.^4^ Here, we re-evaluate the ligand exchange process under more forcing conditions and apply our characterization toolbox to elucidate the cluster structure. Starting from the Zr12-acetate cluster, we stir it with 1.5 equivalents of a new ligand (a carboxylic acid from Fig. 2) before we evaporate all volatile species at 70 °C. Since acetic acid has a reasonably low boiling point, it is readily removed from the reaction mixture, forcing the ligand exchange to proceed to completion (confirmed by the absence of acetate signals in the ^13^C NMR, see Fig. S26†). The clusters are then purified to remove excess high boiling ligand. PDF analysis of the clusters shows that the dimer is obtained for butanoate, hexanoate, octanoate, decanoate, dodecanoate and oleate, see Fig. S27.† The Zr6 monomer is retrieved for methylbutanoate or methylheptanoate, showing that the dimer can be broken up under appropriate conditions and with the right structure-directing ligand. Also, the IR and NMR spectra look identical to the ones from the clusters that are directly synthesized from zirconium propoxide, see Fig. S28 and S29.† We also treated the Zr6-methylbutanoate cluster twice with 3 equivalents of hexanoic acid. Methylbutanoic acid does not evaporate as easily as acetic acid and a higher excess of new ligand was needed. The resulting cluster has the typical IR and NMR signatures of a Zr12 cluster, see Fig. S30 and S31.† Also the HRMS shows similar results compared to the HRMS of bottom up synthesized Zr12-hexanoate, see Fig. S21 and S32.† The results of the ligand exchanges are summarized in Fig. 9.

### 2.6 Hafnium oxo clusters

**Fig. 10:**
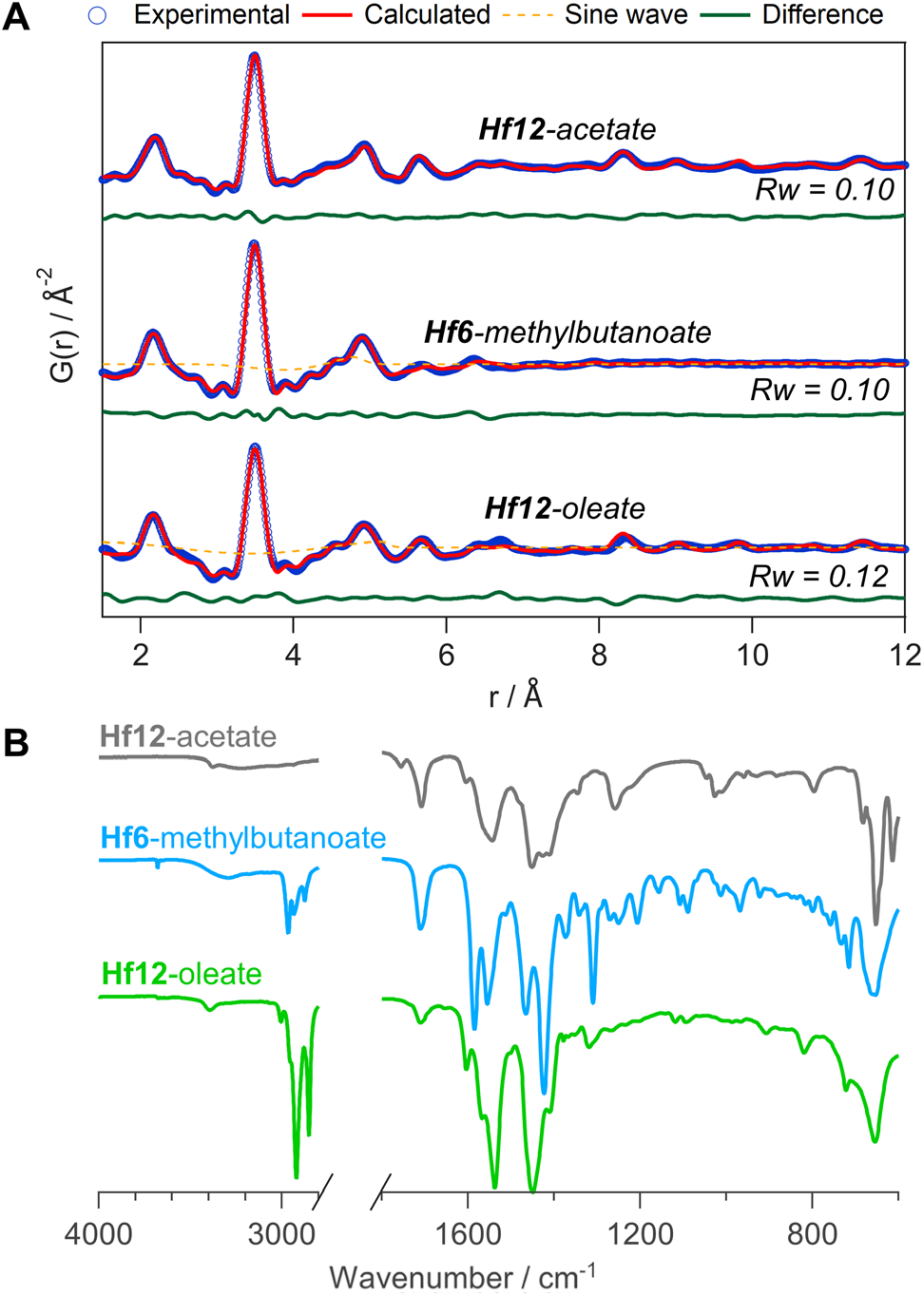
(A) PDF fit for Hf12-acetate, Hf6-methylbutanoate and Hf12-oleate clusters with exponentially dampening sine wave contribution. The refined parameters are indicated in Table S9.† (B) FTIR spectra of the hafnium oxo clusters synthesized *via* bottom up.

To generalize our methodology to hafnium oxo clusters, we reacted hafnium butoxide with acetic acid, methylbutanoic acid, or oleic acid. After purification, the resulting clusters were characterized by PDF, FTIR, TGA, and NMR. The acquisition and refinement of PDF data are more straightforward for the hafnium oxo clusters since hafnium has a higher scattering cross section, see Fig. 10A. All pairs that have at least one hafnium atom are enhanced in intensity, and all other pairs (*e.g.*, C–C) lose relative intensity. Using a structural model of Hf12-acetate (CCDC-604534),^4^ we can readily describe the PDF data of the Hf12-acetate and Hf12-oleate clusters. The goodness of fit is even better than for the respective Zr12 clusters that were refined in an equivalent way, see Fig. 5. In the absence of a Hf6 structure model, we constructed a Hf6 cluster model from the Hf12 structure. This model accurately described the PDF data, confirming that the Hf6-methylbutanoate cluster is a monomer. The hafnium oxo clusters thus follow the same structure-directing rules as the zirconium oxo clusters. Also, the FTIR spectra (Fig. 10B) and NMR spectra (Fig. S33†) show the expected patterns, similar to those in the case of zirconium. Ligand exchanges can be performed from acetate to oleate or methylbutanoate ligands, forming the expected Hf12 or Hf6 cluster respectively, based on FTIR and NMR data (Fig. S34 and S35†). Finally, TGA shows that there is again free carboxylic acid present on top of the charge balancing ligand shell (Fig. S36 and Table S10†).

### 2.7 Catalysis

**Fig. 11:**
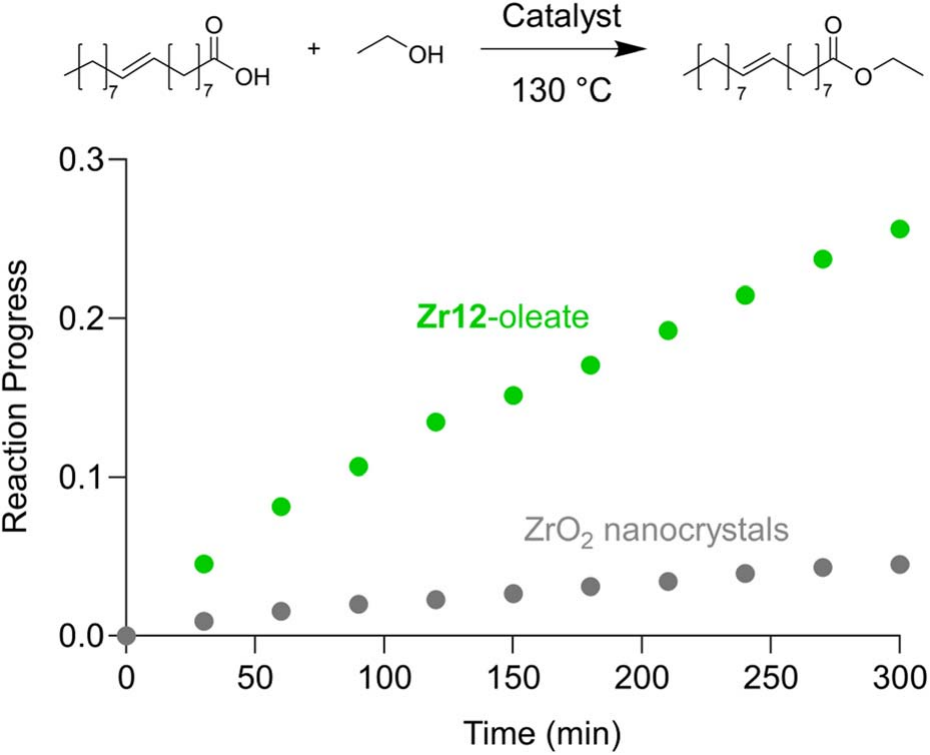
Catalytic esterification of oleic acid with ethanol, catalyzed by either Zr12-oleate (green) or ZrO_2_ nanoparticles (gray). In both cases, 10 mol% Zr, with respect to the carboxylic acid substrate, was added.

Finally, we take the perspective that these fatty acid capped oxo clusters are the smallest conceivable nanocrystal prototypes.^2,30^ While technically not a crystal anymore, such a cluster represents the lower limit of scaling down a ligand capped metal oxide nanocrystal. Hence, it has a maximized surface-to-volume ratio. Maximizing surface area is particularly important in heterogeneous catalysis and we thus hypothesized that oxo clusters (diameter = 0.5 nm) would have superior catalytic performance over metal oxide nanocrystals (diameter = 2–10 nm). To test this, we take the previously reported esterification of oleic acid with ethanol as a catalytic model system.^41^ We synthesized 5.5 nm tetragonal zirconia nanocrystals, and functionalized their surface with oleate ligands,^77^ see also Fig. S37 and S38.†Zr12-oleate clusters are the atomically precise, smallest conceivable prototype of such nanocrystals. Given that both clusters and nanocrystals have the same oleate ligand, the only difference is the inorganic core. In addition, the ligand is identical to the catalytic substrate (oleic acid), thus avoiding competition between the ligand and substrate for the surface sites,^41^ or the formation of side products. To compare the efficiency of the two catalysts, we added in both cases 10 mol% Zr to the reaction mixture. We monitored the reactions for the first 5 hours by taking aliquots and quantifying the ester product *via* NMR, see Fig. 11.

The oxo clusters are clearly more catalytically active than the nanocrystals, confirming our hypothesis. An initial rate analysis shows that the rate of the reaction when using Zr12-acetate is 0.3 mM min^−1^ while for ZrO_2_ nanocrystals it is 0.06 mM min^−1^. The clusters thus feature about a five-fold higher reaction rate, which we relate to a higher surface-to-volume ratio. The cluster has every zirconium atom available at its surface. In contrast, we estimate that 19% (about one fifth) of the zirconium atoms in the nanocrystals are available for catalysis. The other 81% is buried under the surface. This estimation agrees well with the five-fold rate enhancement of the clusters. Zirconium and especially hafnium are less abundant elements so ensuring that all metal atoms are available for catalysis and not lost in the bulk is an important step towards a more sustainable catalyst design.^78^ These results thus show that fatty acid capped oxo clusters are an exciting material class and they can possess advantages over their larger relatives; oxide nanocrystals. One can thus consider (oxo) clusters for many more catalytic reactions that are currently performed with nanocrystal catalysts. As atomically precise nanocrystal prototypes, oxo clusters are also better suited for mechanistic studies compared to nanocrystals which feature polydispersity and a more irregular surface structure.^34,79^

## 3 Conclusion

We have synthesized fatty acid capped zirconium and hafnium oxo clusters with a library of linear and branched carboxylic acids. To prove the structure of the oxo clusters, we used X-ray PDF analysis. We showed that the structure models need to include carboxylate binding groups to obtain excellent refinements. Given the larger scattering power of hafnium, the refinements were even more straightforward for the hafnium oxo clusters. We were thus able to prove the structure of our fatty acid capped oxo clusters, despite the fact that they cannot be crystallized. Given the lack of crystallization, we developed purification protocols akin to the ones for colloidal nanocrystals, reinforcing the notion that these fatty acid capped oxo clusters are atomically precise nanocrystal prototypes, of the smallest conceivable scale.

We further comprehensively characterized the oxo clusters by FTIR, NMR, TGA and ESI-HR-MS. We find that the cationic cluster cores are charge balanced by chelating and bridging carboxylate ligands and that a small excess of carboxylic acid is hydrogen bonded to the cluster core. Using our characterization tools, we studied carboxylate for carboxylate ligand exchange and found that the Zr6/Hf6 monomer and the Zr12/Hf12 dimer structure can be converted into one another, depending on the structure of the final ligand. Finally, we compared the catalytic performance of the clusters with that of larger oxide nanocrystals. We found that, for the same amount of zirconium added, the oxo clusters show a five times higher esterification rate than oxide nanocrystals, due to their higher surface area. Since the oxo clusters are the limit of downscaling oxide nanocrystals, we thus propose them here as appealing materials, or at least as atomically precise model systems.

## 4 Experimental

### 4.1 Materials

Zirconium propoxide (70 w% in 1-propanol) and hafnium butoxide (99%) was provided by Sigma-Aldrich and stored in a Straus flask upon arrival. Acetic acid (>99%) was purchased from Sigma-Aldrich and vacuum distilled after which it was stored in a Schlenk flask. Zirconium isopropoxide isopropanol complex (99.9%), propionic acid (>99.5%), butyric acid (99%), hexanoic acid (99%), octanoic acid (99%), decanoic acid (>99.5%), dodecanoic acid (98%), oleic acid (90%), methyl-butyric acid (98%), methyl-heptanoic acid (>98.5%), pivalic acid (99%) and benzyl alcohol (anhydrous, 99.8%) were bought from Sigma-Aldrich and used without any further purification. Acetone, diethylether (BHT stabilized) and dichloromethane (DCM) were bought from Biosolve and used without any further purification. HPLC grade acetonitrile (ACN) was bought from VWR and used without any further purification. All yields reported here are calculated without any co-precipitated molecules unless otherwise specified. Centrifugation was always performed at 5000 rcf for 3 minutes, unless otherwise specified. After purification all clusters are stored in a desiccator.

### 4.2 Zr12-acetate synthesis

A 20 mL vial was equipped with a septum and cycled three times between argon and vacuum. Zirconium propoxide (2.25 mL, 5 mmol, 1 eq.) was added to the vial, together with dry DCM (5.463 mL). Under stirring, distilled acetic acid (2.287 mL, 40 mmol, 8 eq.) was injected, reaching a total reaction volume of 10 mL and thus a zirconium concentration of 0.5 M. After 12 hours at room temperature (19–24 °C), the crystalline powder is isolated by filtration and further washed with 50 mL of a DCM : acetic acid mixture (4 : 1). Finally, the white powder was dried overnight under high vacuum. The powder was stored in a desiccator. The cluster was obtained as a white solid, with a 98% yield, calculated based on the molecular formula: Zr_12_O_8_(OH)_8_(CH_3_COO)_24_·6CH_3_COOH·3.5DCM).^4^

### 4.3 Zr12-Propionate synthesis

Zirconium propoxide (2.35 mL, 5.22 mmol, 1 eq.) was mixed with propionic acid (3.13 mL, 41.8 mmol, 8 eq.). After overnight reaction the solid was washed with a 1-propanol : propionic acid mixture (ratio 1 : 3.4). After washing, the solid was dried for 8 hours on a Schlenk line. Zr12-propionate was obtained as a white solid with a yield of 51%, calculated based on the molecular formula: Zr_12_O_8_(OH)_8_(CH_3_CH_2_COO)_24_·6CH_3_CH_2_COOH).^4^

### 4.4 Zr12-Butanoate, hexanoate and octanoate

For butanoate, hexanoate and octanoate capped clusters, zirconium propoxide (2.25 mL, 5 mmol, 1 eq.) was first mixed with DCM, and afterwards 8 equivalents of acid was added. The amount of DCM is adjusted to maintain a Zr concentration of 0.5 M. After stirring for 24 hours at 30 °C the clusters were purified. The butanoate capped cluster was purified by evaporating the solvent using the Schlenk line after which a white waxy solid was obtained. Acetonitrile (15 mL) was added and the clusters were macerated overnight. After centrifugation for 4 minutes at 5000 rcf the supernatant was discarded and the clusters were dried on the Schlenk line for 24 h yielding a white solid. For hexanoate and octanoate clusters the solution was divided over 2 large centrifuge tubes (±5 mL reaction mixture in each) and acetonitrile (10 mL) was added to each centrifuge tube in order to precipitate the clusters. *Via* centrifugation (5000 rcf, 4 min), the cluster (viscous oil) was separated from the supernatant and both oils were redissolved in DCM (1 mL). To each tube 6 mL acetonitrile was added to precipitate the cluster. This last step was repeated twice and finally, the clusters were dried overnight under vacuum. Zr12-hexanoate was obtained as a waxy solid and Zr12-octanoate was obtained as a viscous liquid. Zr12-butanoate was obtained with a yield of 49%. Zr12-hexanoate was obtained as a waxy solid with a yield of 71%. Zr12-octanoate was obtained as a viscous oil with a yield of 52%.

### 4.5 Zr12-Decanoate, dodecanoate and oleate

For decanoic and dodecanoic acid, zirconium propoxide (2.25 mL, 5 mmol, 1 eq.) was mixed with 8 equivalents of acid and diluted with DCM to 0.33 M and 0.25 M respectively in Zr. Decanoic and dodecanoic acids are solids and therefore the concentration had to be lowered in order to dissolve these acids. For oleic acid (which is a liquid), the volume is already 15 mL without DCM. Therefore, no DCM was added and a 40 mL flask was used. After stirring for 24 hours at 30 °C, DCM was evaporated using the Schlenk line for both Zr12-decanoate and Zr12-dodecanoate, while the oleate sample can be purified as such. The solution for each cluster was divided over 2 centrifuge tubes and ACN (10 mL per tube) was added to precipitate the clusters. *Via* centrifugation (5000 rcf, 4 min), the cluster precipitate was separated from the supernatant and redissolved in DCM (1 mL). To this 6 mL acetone was added to precipitate the cluster. This last step was repeated twice and finally, the clusters were dried under vacuum. Zr12-decanoate and Zr12-dodecanoate were obtained as waxy solids with a yield of 80% and 93% respectively. Finally, Zr12-oleate was obtained as a viscous oil with a yield of 83%.

### 4.6 Zr6-Methylbutanoate and methylheptanoate

Zirconium propoxide (2.25 mL, 5 mmol, 1 eq.) was mixed with 8 equivalents of acid and diluted with DCM to 0.5 M in Zr. After stirring for 48 hours at 30 °C, the solution was dried under vacuum and dissolved again in 1 mL DCM. Afterwards, the solution is divided over 2 centrifuge tubes and acetonitrile (4 mL per tube) was added to precipitate the clusters. *Via* centrifugation (5000 rcf, 5 min), the cluster precipitate was separated from the supernatant and redissolved in DCM (1 mL each). To both tubes acetonitrile (double the volume) was added to precipitate the cluster. This last step was repeated twice and finally, the clusters were dried overnight under vacuum. Zr6-methylbutanoate was obtained as a white solid with a yield of 93%. Zr6-methylheptanoate was obtained as a viscous oil with a yield of 55%.

### 4.7 Zr12-Acetate ligand exchange

The Zr12-acetate cluster (200 mg, 1.4 mmol acetate, 1 eq.) was weighed into a 20 mL vial. The incoming carboxylic acid (1.5 equivalents) was added together with 1 mL of DCM and stirred for 60 min, after which a clear solution was obtained. Subsequently, the solution was further stirred under vacuum at 70 °C for 1 hour. The sample transforms from a heterogeneous mixture into a viscous liquid. 1 purification step was performed as described above for the respective cluster. Yields: butyric acid = 82%, methylbutyric acid = 41%, hexanoic acid = 97.2%, octanoic acid = 95%, and oleic acid = 100%.

### 4.8 Zr6-Methylbutanoate ligand exchange

The Zr6-methylbutanoate cluster (200 mg, 1.27 mmol methylbutanoate, 1 eq.) was weighed into a 20 mL vial. 2 mL DCM together with 3 equivalents of hexanoic acid were added and the solution was stirred for 1 hour at room temperature. Afterwards, the solution is dried under vacuum for 1 hour at 70 °C. One purification cycle was performed by dissolving the sample in 0.5 mL DCM and precipitating it by adding 2 mL ACN. This turbid solution was centrifuged at 5000 rcf for 4 minutes. The whole process was repeated once to ensure full exchange. After the second exchange step the sample was purified twice as described above. Zr12-hexanoate was obtained as a viscous liquid with a yield of 56.5%.

### 4.9 Hf12-Acetate, oleate and methylbutanoate

Hafnium *n*-butoxide (1.9 mL, 5 mmol, 1 eq.) was mixed with 8 equivalents of acetic, oleic or methylbutyric acid. Prior to mixing dry DCM was added to the hafnium precursor in order to set [Hf] = 0.5 M in the whole reaction mixture. Hf12-acetate and Hf12-oleate were reacted for 24 h at 30 °C. For Hf12-oleate no dry DCM was added. The Hf6-methylbutanoate synthesis was kept at 30 °C for 48 h. Purification was performed according to the zirconium clusters for the respective acids. Hf12-acetate and Hf6-methylbutanoate were obtained as white solids with yields of 100% and 50%, respectively. Hf12-oleate was obtained as a viscous oil with a yield of 37%.

### 4.10 Hf12-Acetate ligand exchange

The Hf12-acetate cluster (200 mg, 1.26 mmol acetate, 1 eq.) was weighed into a 20 mL vial. 1.5 equivalents (1.89 mmol) of the new carboxylic acid, 597 μL oleic acid or 301 μL methylheptanoic acid, was added together with 1 mL of DCM and stirred for 60 min, after which a clear solution was obtained. Subsequently, the solution was further stirred under vacuum at 70 °C for 1 hour. The sample transforms from a heterogeneous mixture into a viscous liquid. Purification was performed as described above for the respective cluster. The exchanged clusters were obtained as a viscous oil with a yield of 70% for the oleate exchanged cluster and 37% for the methylheptanoic acid exchanged clusters.

### 4.11 Nanocrystal synthesis

The zirconia nanocrystals were synthesized according to Garnweitner *et al.* with few modifications.^77^ Zr(OiPr)_4_·iPrOH (6.6 g, 17 mmol) was mixed with benzyl alcohol (60 mL, 580 mmol) in a Parr bomb inside a nitrogen filled glovebox. The Parr bomb was heated for 48 h at 210 °C. After the reaction, a white powder was obtained *via* centrifugation. This was washed with 60 mL diethyl ether followed by centrifugation three times (in 4 centrifuge tubes with 15 mL each). The white solid is dispersed in 40 mL toluene (4 centrifuge tubes of 10 mL each). To each centrifuge tube oleic acid was added (600 μL, 1.9 mmol) to functionalize the nanocrystal surface and the centrifuge tubes were stirred and sonicated for 30 minutes. This procedure resulted in a cloudy solution of nanocrystals. The insolubles were removed by centrifugation and a transparent solution of the soluble nanocrystals in the supernatant was collected in 2 centrifuge tubes and the nanocrystals were precipitated using a 1 : 1 ratio of nanocrystal solution and acetone. The collected nanocrystals were then purified *via* three precipitation/redissolving cycles; the nanocrystals in 10 mL toluene were split off into two centrifuge tubes and precipitated by adding 15 mL acetone to each of the two tubes. The nanocrystals were obtained as a white solid with a yield of 43%.

### 4.12 Catalytic experiments

10 mol% (in Zr) of the catalyst was dispersed in a 4 mL solution of 200 mM oleic acid and 400 mM EtOH in o-dichlorobenzene. 10 mol% Zr corresponds to 54.36 mg of the Zr12-oleate cluster (0.0067 mmol clusters or 0.08 mmol Zr) and 9.86 mg ZrO_2_ (0.08 mmol Zr). The reaction mixture is then heated to 130 °C for 5 h in a closed vial. Aliquots are taken every 30 min.

### 4.13 General instrumentation

Nuclear magnetic resonance (NMR) measurements were recorded at 298 K on a Bruker UltraShield 500 spectrometer operating at a frequency of 500.13 MHz. The IR spectra were recorded on a PerklinElmer spectrum 2 ATR-FTIR with a diamond crystal.

### 4.14 Mass spectrometry

ESI mass spectra were acquired using a Bruker maXis4G or maXis II high resolution mass spectrometer equipped with an electronspray ionization source. The cluster was dissolved in a mixture of ACN : DCM, in which the amount of ACN was maximized while avoiding precipitation of the clusters. The samples were directly introduced into the instrument at a rate of 6 μL min^−1^ using a syringe pump. The heated capillary temperature was 200 °C and the capillary voltage was 3.6 kV. The samples were analyzed in positive ion mode. DataAnalysis 4.4 from Bruker was used to process the raw data.

### 4.15 Synchrotron X-ray total scattering experiments

The samples were prepared in a 1 mm polyamide kapton tube and were measured either at beamline 11-ID-BM at the Advanced Photon Source, Argonne National Laboratory, USA or at beamline P21.1 at DESY in Hamburg, Germany. X-ray total scattering data were collected at room temperature in rapid acquisition mode, using a PerkinElmer digital X-ray flat panel amorphous silicon detector (2048 × 2048 pixels and 200 × 200 μm pixel size) with a sample-to-detector distance of 180 mm (11-ID-BM) or 380 mm (P21.1). The incident wavelength of the X-rays was *λ* = 0.2110 Å (11-ID-BM) or 0.1220 Å (P21.1). Calibration of the experimental setup was performed using a Ni standard.

### 4.16 Analysis of synchrotron X-ray total scattering data

Raw 2D data were corrected for geometrical effects and polarization, and then azimuthally integrated to produce 1D scattering intensities *versus* the magnitude of the momentum transfer *Q* (where *Q* = 4π sin *θ*/*λ* for elastic scattering) using pyFAI and xpdtools.^80,81^ The program xPDFsuite with PDFgetX3 was used to perform the background subtraction, further corrections, and normalization to obtain the reduced total scattering structure function *F*(*Q*), and Fourier transformation to obtain the pair distribution function (PDF), *G*(*r*).^82,83^ For data reduction, the following parameters were used after proper background subtraction: *Q*_min_ = 0.8 Å^−1^, *Q*_max_ = 22 Å^−1^, and *R*_poly_ = 0.9 Å. We used the actual chemical composition of each cluster (inorganic core + charge balancing ligands) for data reduction. Modeling and fitting were carried out using Diffpy-CMI.^84^ The Debye scattering equation was used to generate the calculated PDF from discrete structure models. The structure models are supplied as *xyz* files in the ESI.† The refinements were carried out by refining the scale factor, isotropic atomic displacement parameters (Uiso), and delta2 (coefficient for the 1/*r*^2^ contribution to the peak sharpening). The exponentially dampening sine-wave contribution was calculated according to the following equation:
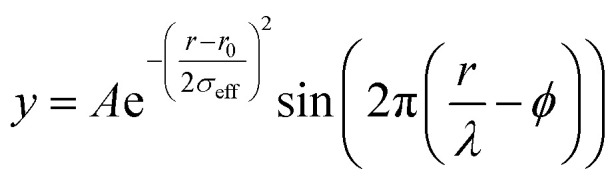
*A* – amplitude of oscillation, *r* – the distance in the PDF, *λ* – wavelength, *ϕ* – phase shift, *σ* – effective dampening with *σ*_eff_ = *σ*/*a* for *r* < *r*_0_ and *σ*_eff_ = *σ* × *a* for *r* > *r*_0_, *a* is the asymmetry parameter. and *r*_0_ is not a physical parameter in real space and is used to describe different dampening behaviors.^64^

## Data availability

The data underlying the figures is available on the Zenodo platform, DOI: 10.5281/zenodo.7373015. In the ESI,† further characterization *via* PDF, NMR, MS and FTIR is available. The ESI† also contains the structure models that we used for the PDF refinements.

## Author contributions

D. Van den Eynden: synthetic and analytic methodology, investigation, writing – original draft, visualization. R. Pokratath: PDF analysis, TGA data acquisition, writing – original draft, visualization. J. Pulparayil Mathew: validation, investigation (catalysis). E. Goossens: TGA data acquisition. K. De Buysser: supervision. J. De Roo: conceptualization, supervision, writing – review and editing, visualization.

## Conflicts of interest

There are no conflicts to declare.

## Supplementary Material

SC-014-D2SC05037D-s001

SC-014-D2SC05037D-s002
