# Supplementary material for: Fatty acid capped, metal oxo clusters as the smallest conceivable nanocrystal prototypes
Source: Chem Sci. 2022 Dec 8;14(3):573–85. doi: 10.1039/d2sc05037d (PMC9847641; doi:10.1039/d2sc05037d)
Supplement: SC-014-D2SC05037D-s002 [file SC-014-D2SC05037D-s002.pdf]

Electronic Supplementary Material (ESI) for Chemical Science.
This journal is © The Royal Society of Chemistry 2022

Supporting Information: Fatty acid capped, metal oxo clusters as smallest conceivable nanocrystal prototypes

Dietger Van den Eynden,[†,‡] Rohan Pokratath,[†] Jikson Pulparayil Mathew,[†] Eline Goossens,[†,‡] Klaartje De Buysser,[‡] and Jonathan De Roo[*,†]

†*Department of Chemistry, University of Basel, Mattenstrasse 24a, 4058 Basel, Switzerland*

‡*Department of Chemistry, university of Ghent, Krijgslaan 281, 9000 Ghent, Belgium*

E-mail: Jonathan.DeRoo@unibas.ch

Contents

1 Cluster synthesis **S-2**

2 Pair Distribution Function analysis **S-3**

3 The organic ligand shell **S-11**

4 NMR analysis **S-16**

5 HR-MS **S-18**

6 Clusters synthesized by ligand exchange **S-23**

7 Hafnium oxo clusters **S-27**

8 Zirconium oxide nanocrystals **S-31**

References **S-32**

1 Cluster synthesis

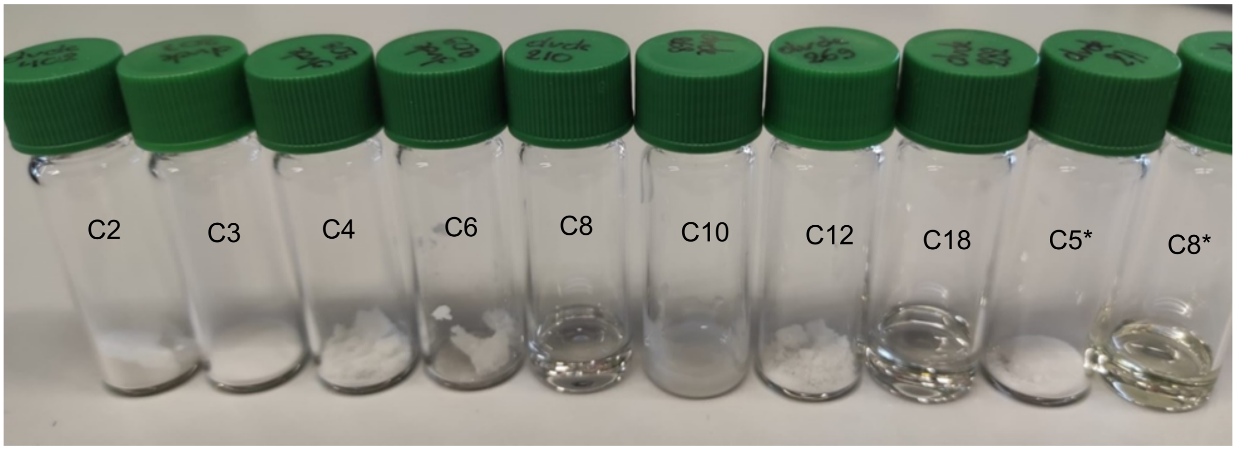

Figure S1: Overview aggregation state fatty acid capped clusters. C2-C18 = **Zr12**-acetate to **Zr12**-oleate, C5* = **Zr6**-methylbutanoate and C8* = **Zr6**-methylheptanoate.

2 Pair Distribution Function analysis

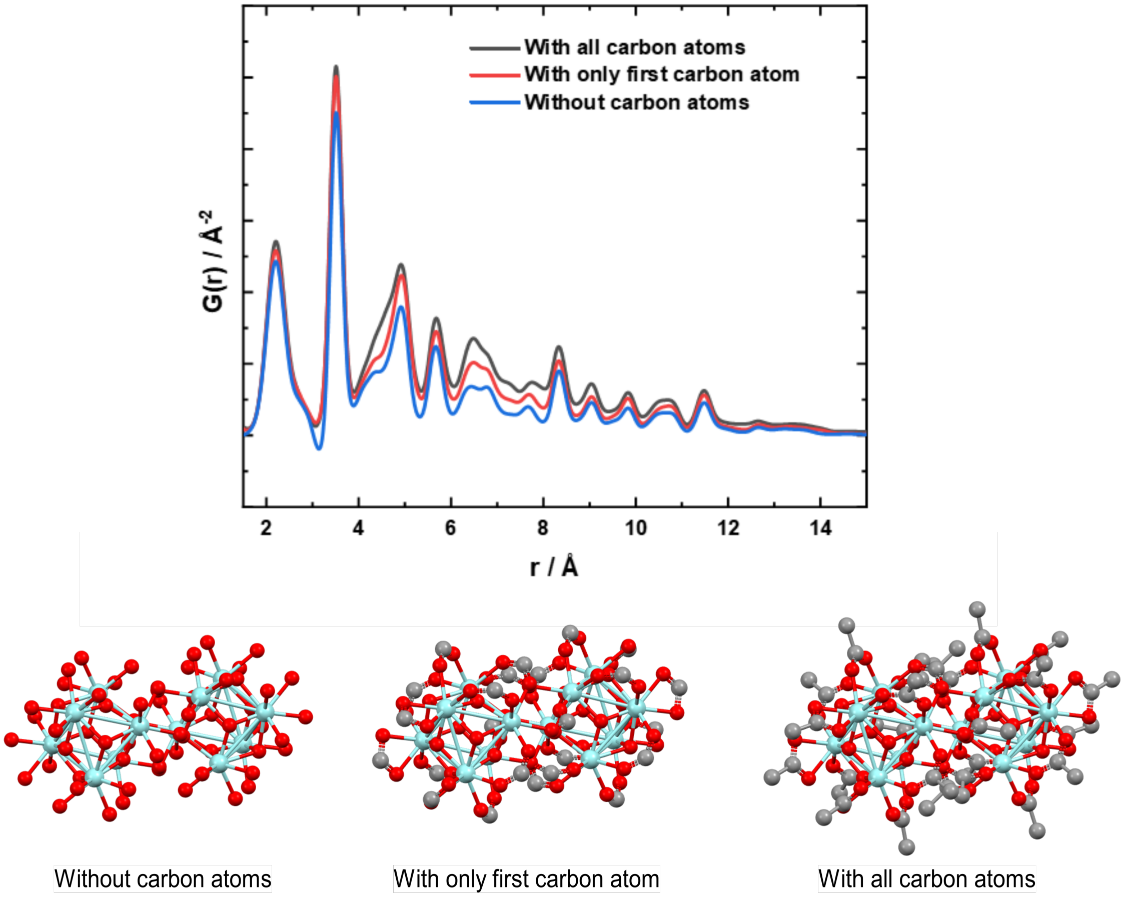

Figure S2: Theoretically calculated X-ray PDF for the **Zr12**-acetate structure, which features bridging and chelating acetate ligands.[S1] The hydrogen atoms were not included in any model since hydrogen atoms have an extremely low scattering cross section for X-rays. We used the following atomic displacement parameters (ADP) for the calculation. Zr: 0.007 Å^2, O: 0.02 Å^2, C: 0.02 Å^2. The structures used for calculations are shown.

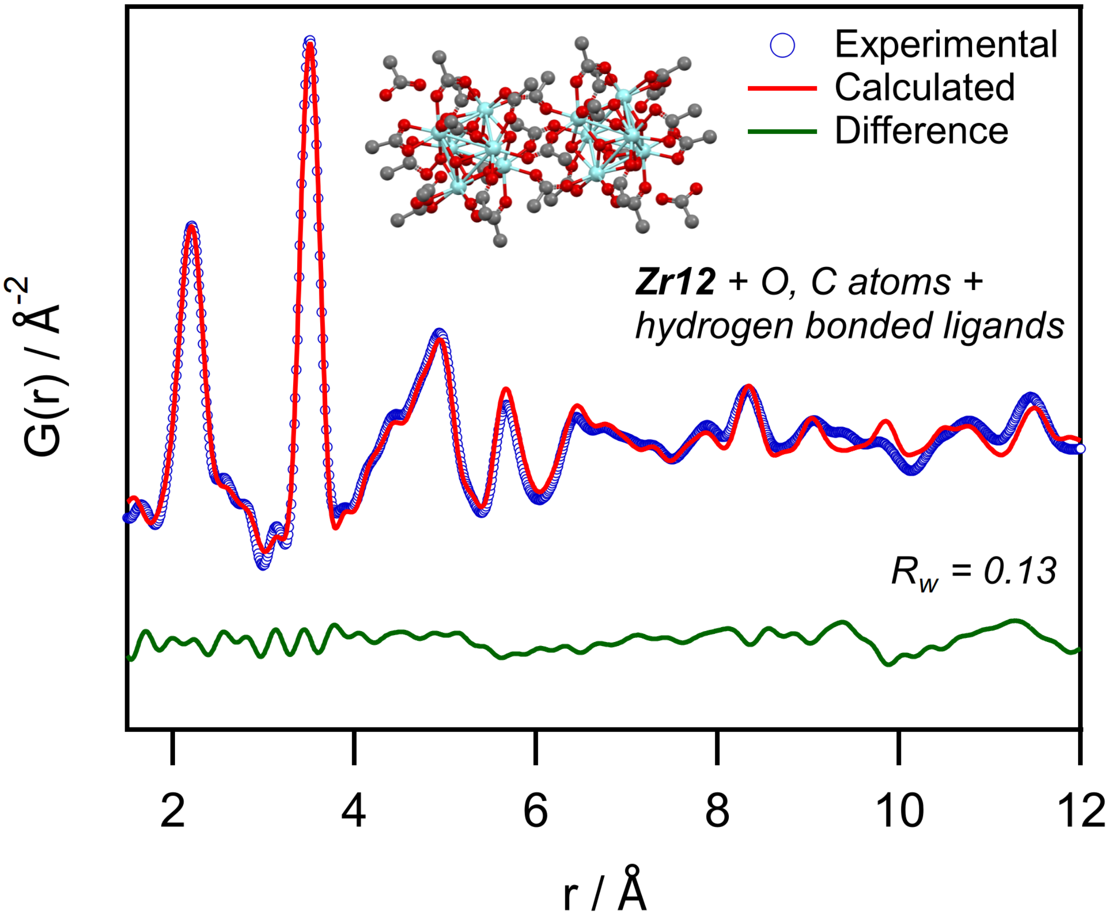

Figure S3: PDF refinement of the **Zr12**-acetate PDF with the **Zr12**-acetate cluster model, including the hydrogen bonded ligands. The refined parameters are given in Table S1.

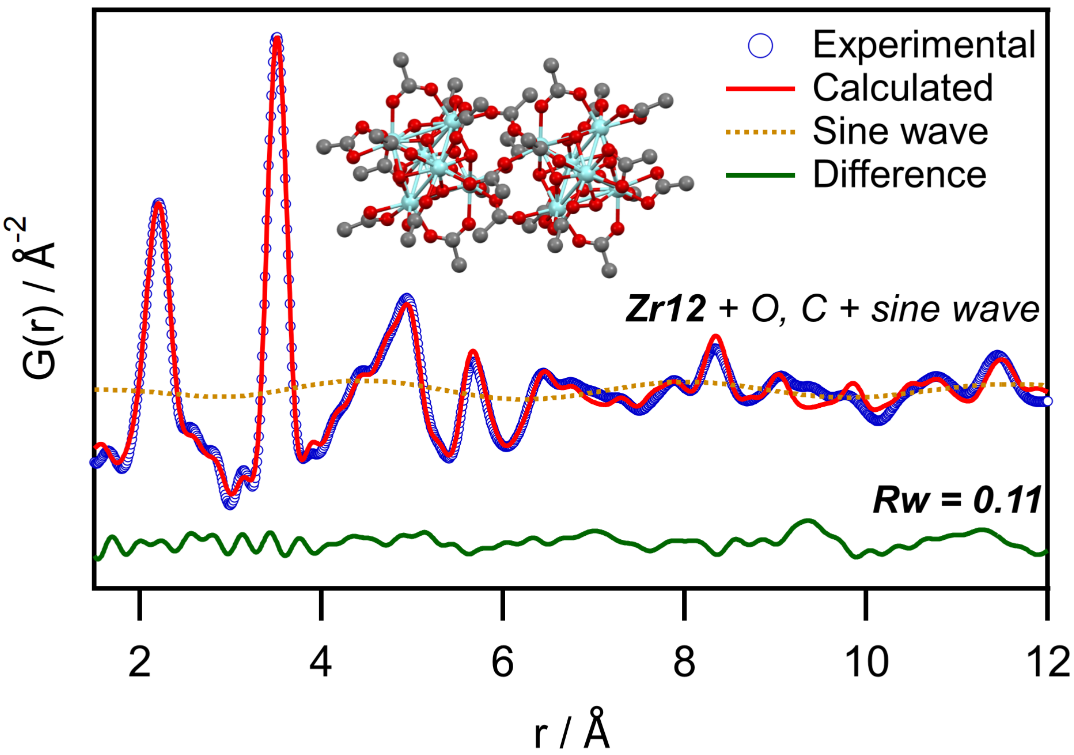

Figure S4: PDF refinement of the **Zr12**-acetate PDF with the **Zr12**-acetate cluster model, including an exponentially dampening sine wave contribution. The refined parameters are given in Table S1.

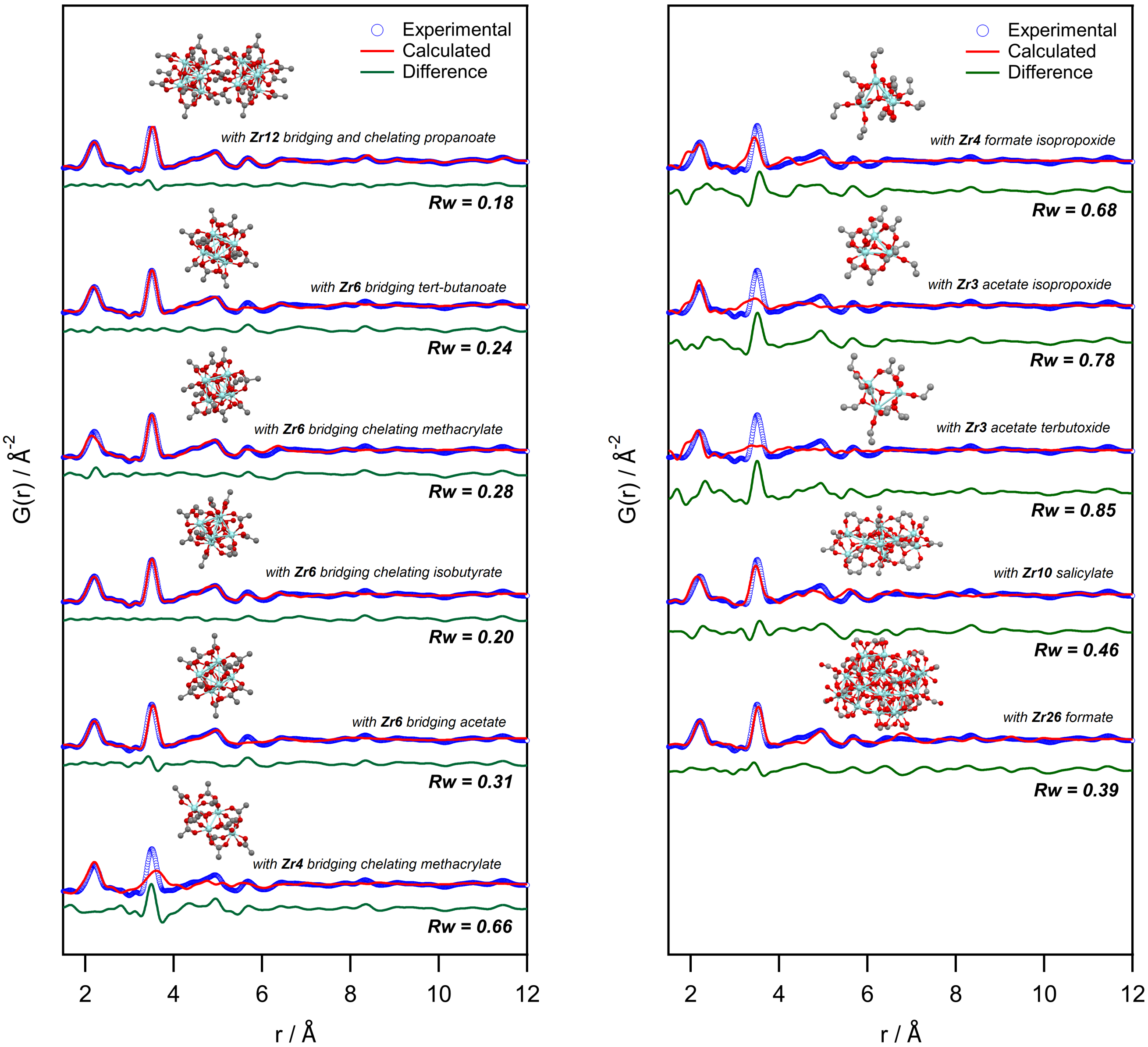

Figure S5: PDF refinement of the **Zr12**-acetate PDF with various cluster structures reported in literature (see main text). For each of the structural models, we removed the excess carbon atoms to arrive at a model with acetate ligands. The refined parameters are given in Table S2.

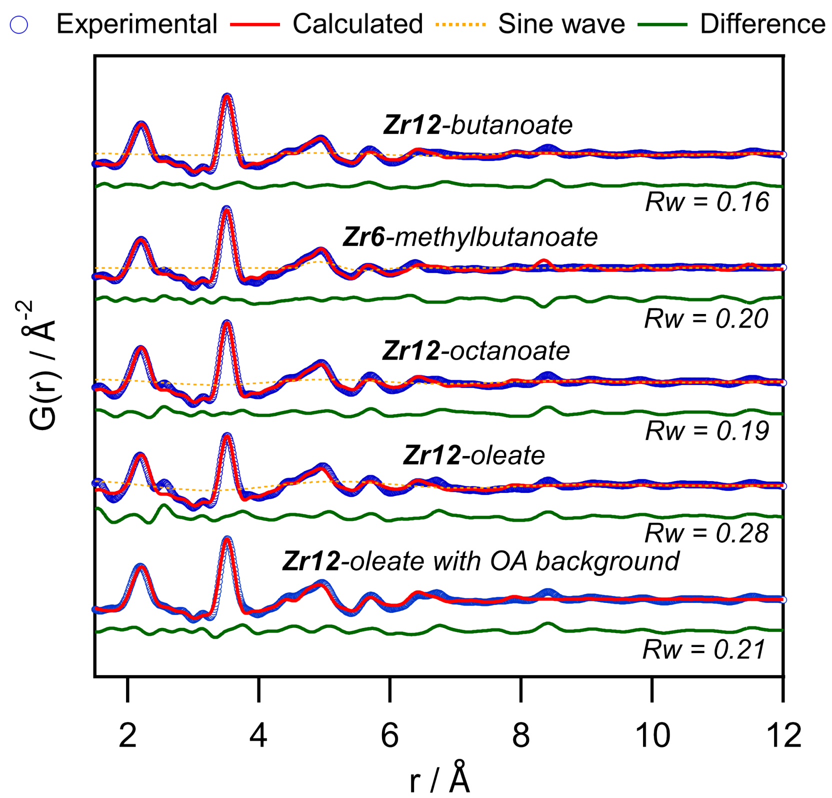

Figure S6: PDF refinement for **Zr12**-butanoate (using the **Zr6**-acetate structural model), **Zr6**-methylbutanoate (using the **Zr12**-acetate structural model), **Zr12**-octanoate (using the **Zr6**-acetate structural model), and **Zr12**-oleate (using the **Zr6**-acetate structural model). The contribution of the exponentially dampening sine wave is shown (orange dotted lines). The refined parameters are given in Table S4.

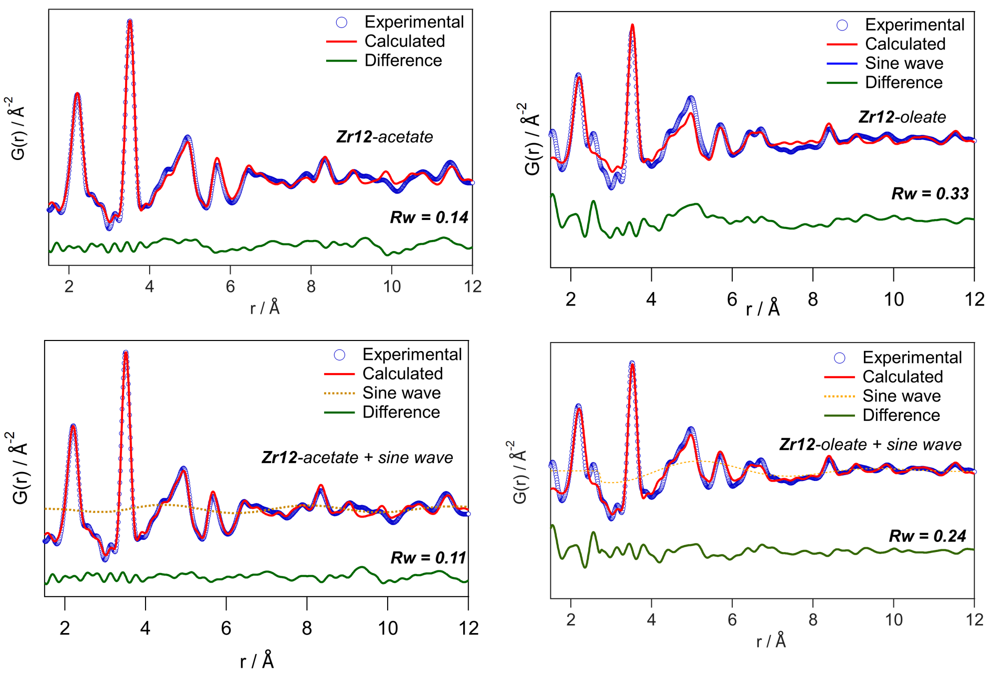

Figure S7: PDF fit for **Zr12**-acetate/oleate cluster with **Zr12**-acetate model without/without the exponentially dampening sinusoidal contribution. The refined parameters are given in Table S5.

Table S1: Refined parameters after fitting our synthesized **Zr12**-acetate cluster with various models, see Figure 2, Figure S3 and S4. All the models were derived from the crystal structure of **Zr12**-acetate,[S1] and in all models, the hydrogen atoms were removed. Relative Amplitude = Amplitude / Scale.

**Model**	**Zr6** core	**Zr12** core	**Zr12** with all O	**Zr12** with all O and C	**Zr12** with H-bonded ligands	**Zr12** with sine wave
**Scale**	0.95	0.94	0.66	0.60	0.61	0.60
**Uiso Zr (Å^2)**	0.004	0.004	0.005	0.004	0.004	0.004
**Uiso O (Å^2)**	0.005	0.005	0.011	0.011	0.011	0.011
**Uiso C (Å^2)**				0.013	0.013	0.013
**delta2 (Å^2)**	2	2	2	2.04	1.96	2.10
**Rw**	0.48	0.46	0.23	0.14	0.13	0.11
**Amplitude (A)**						-0.157
**Relative Amplitude**						0.261
**wasyn**						1.832
λ						3.616
ϕ						0.474
θ						4.815
**wsig**						2.701

Table S2: Refined parameters after fitting our synthesized **Zr12**-acetate cluster with various models, see Figure S5. In all models, some carbon atoms were removed in order to form a structure equivalent to an acetate capped cluster and all hydrogen atoms were removed.

Model	**Zr4** bridging, chelating methacrylate	**Zr6** bridging acetate	**Zr6** bridging, chelating isobutanoate	**Zr6** bridging, chelating methacrylate	**Zr6** bridging butanoate	**Zr12** bridging, chelating propanoate
**Scale**	1.01	0.58	0.59	0.55	0.59	0.60
**Uiso Zr (Å^2)**	0.027	0.004	0.004	0.005	0.005	0.004
**Uiso O (Å^2)**	-0.014	0.003	0.011	0.029	0.020	0.011
**Uiso C (Å^2)**	0.022	0.011	0.017	0.035	0.024	0.013
**delta2 (Å^2)**	-5.88	1.19	1.14	2.85	3.55	2.10
**Rw**	0.66	0.20	0.31	0.24	0.18	0.11
**Amplitude (A)**	-0.148	-0.209	-0.220	-0.209	-0.156	-0.157
**wasyn**	2.04	2.275	0.058	9.917	1.438	1.832
λ	2.949	3.679	3.97	3.541	3.622	3.616
ϕ	1.009	0.432	0.247	0.522	0.462	0.474
θ	4.609	4.406	0.115	2.979	5.181	4.815
**wsig**	2.413	3.145	0.474	0.904	3.038	2.701
**Model**	**Zr4** formate isopropoxide	**Zr3** acetate isopropoxide	**Zr3** acetate *t*-butoxide	**Zr10** salicylate	**Zr26** formate	....
**Scale**	0.76	0.85	0.72	0.64	0.50	
**Uiso Zr (Å^2)**	0.005	0.021	0.036	0.005	0.006	
**Uiso O (Å^2)**	0.003	-0.013	-0.586	-0.0007	0.009	
**Uiso C (Å^2)**	0.728	0.183	0.728	0.190	0.052	
**delta2 (Å^2)**	1.98	1.94	4.08	1.86	4.24	
**Rw**	0.68	0.78	0.84	0.46	0.39	
**Amplitude (A)**	-0.198	-0.356	-0.247	-0.369	-0.254	
**wasyn**	5.021	4.995	0.168	4.716	4.087	
λ	3.908	3.545	3.800	3.647	3.244	
ϕ	0.857	0.578	0.404	0.897	0.475	
θ	3.689	2.487	0.558	3.678	1.478	
**wsig**	1.985	0.587	2.669	1.119	1.002	

Table S3: Refined parameters after fitting various zirconium oxo clusters with different ligands, using the **Zr12**-acetate, **Zr12**-propionate or the /**Zr6**-acetate model. Relative Amplitude = Amplitude / Scale.

	**Zr12**-butanoate	**Zr6**-methylbutanoate	**Zr12**-octanoate	**Zr12**-oleate	**Zr12**-oleate (background subtracted)
**Model**	**Zr12**-acetate	**Zr6**-acetate	**Zr12**-acetate	**Zr12**-propanoate	**Zr12**-propanoate
**Scale**	0.63	0.56	0.50	0.29	0.64
**Uiso Zr (Å^2)**	0.004	0.003	0.004	0.005	0.005
**Uiso O (Å^2)**	0.010	0.011	0.013	0.019	0.017
**Uiso C (Å^2)**	0.011	0.014	0.010	0.018	0.018
**delta2 (Å^2)**	2.04	1.54	2.73	3.39	2.87
**Rw**	0.13	0.13	0.16	0.24	0.15
**Amplitude (A)**	0.165	0.349	0.331	0.295	
**Relative Amplitude**	0.262	0.623	0.662	1.01	
**wasyn**	-0.077	5.163	-4.540	-10.262	
λ	4.290	4.872	3.574	4.532	
ϕ	-1.024	4.677	-4.833	-5.087	
θ	5.191	4.623	2.932	2.767	
**wsig**	1.173	0.175	-0.332	-0.258	

Table S4: Refined parameters after fitting various zirconium oxo clusters with different ligands, using the opposite model of Table S3.

	**Zr12**-butanoate	**Zr6**-methylbutanoate	**Zr12**-octanoate	**Zr12**-oleate	**Zr12**-oleate (background subtracted)
**Model**	**Zr6**-acetate	**Zr12**-acetate	**Zr6**-acetate	**Zr6**-acetate	**Zr6**-acetate
**Scale**	0.64	0.53	0.51	0.30	0.66
**Uiso Zr (Å^2)**	0.004	0.004	0.004	0.004	0.004
**Uiso O (Å^2)**	0.005	0.023	0.007	0.006	0.004
**Uiso C (Å^2)**	0.011	0.017	0.010	0.008	0.008
**delta2 (Å^2)**	2.64	3.37	1.57	1.64	2.13
**Rw**	0.16	0.20	0.19	0.28	0.21
**Amplitude (A)**	-0.158	0.662	0.231	0.259	
**wasyn**	1.027	-5.766	1.174	0.426	
λ	3.553	1.573	3.634	3.996	
ϕ	0.623	-4.112	-0.842	5.066	
θ	6.472	4.602	3.309	3.457	
**wsig**	3.061	0.091	2.267	-5.372	

Table S5: Refined parameters after fitting **Zr12**-oleate cluster with **Zr12**-propionate model without the exponentially dampening sinusoidal contribution.

	**Zr12**-oleate
**Scale**	0.29
**Uiso Zr (Å^2)**	0.005
**Uiso O (Å^2)**	0.018
**Uiso C (Å^2)**	0.016
**delta2 (Å^2)**	3.32
**Rw**	0.33

Table S6: Comparison of the single crystal distance data of different clusters. For methacrylic acid, only the intracluster distances could be compared as this forms a monomer.[S1]

**Distance**	**Zr12**-acetate (Å)	**Zr12**-propionate (Å)	**Zr6**-methacrylate (Å)
Zr-O	2.03-2.40	2.03-2.41	2.05-2.41
Intra Zr-Zr 1	3.45-3.59	3.47-3.60	3.48-3.55
Intra Zr-Zr 2	4.95-4.97	4.97-5.00	4.96
Inter Zr-Zr 1	5.59-5.68	5.68-5.70	
Inter Zr-Zr 2	8.27-8.61	8.36-8.43	
Inter Zr-Zr 3	11.26-11.96	11.27-12.00	

3 The organic ligand shell

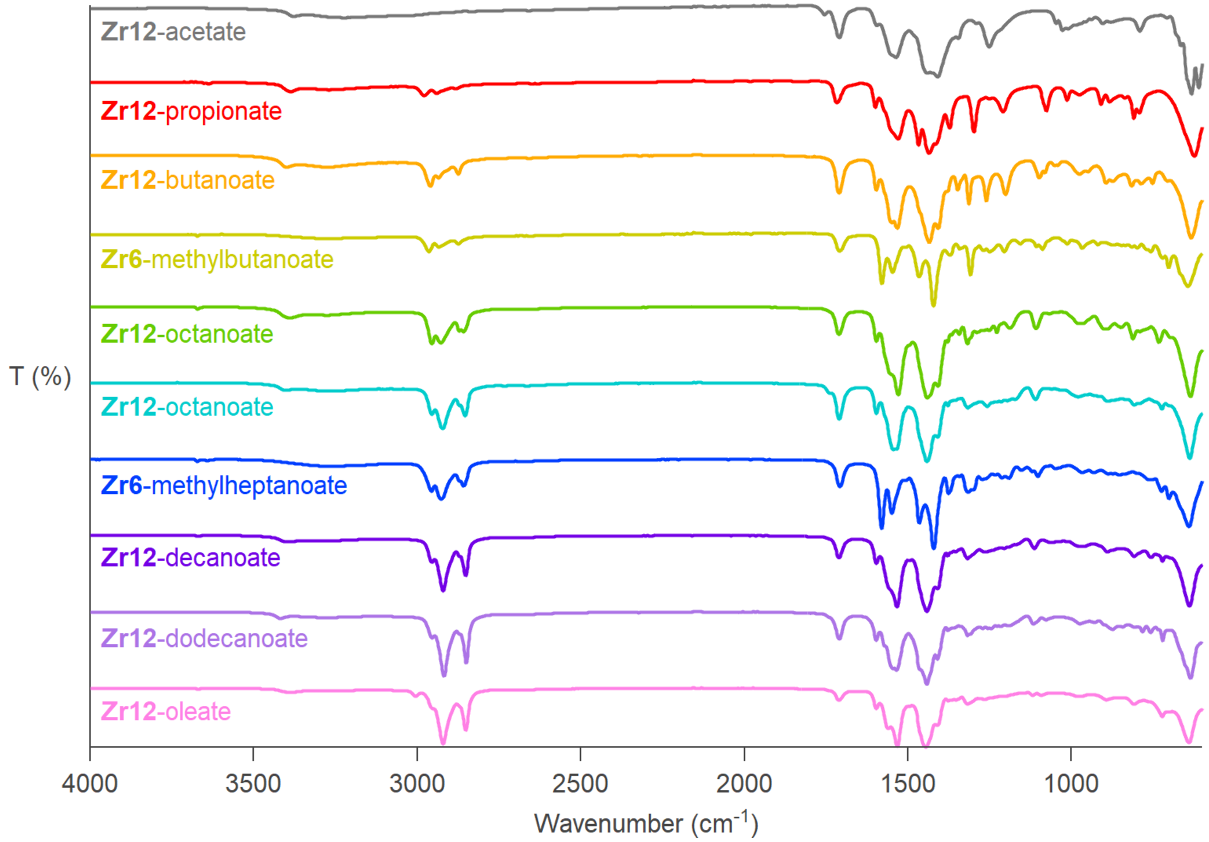

Figure S8: IR of the all bottom up synthesized clusters.

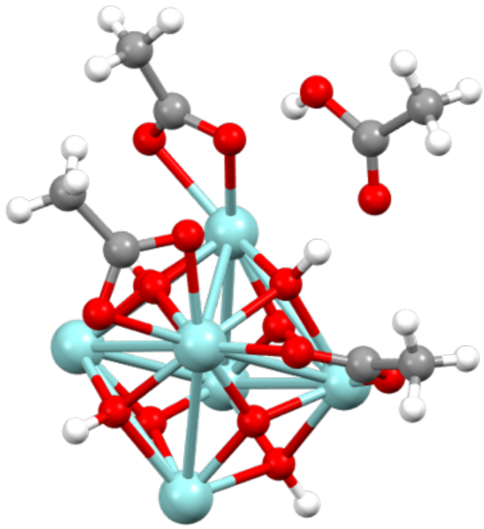

Figure S9: Zoom of the **Zr12**-acetate cluster crystal structure to display the H-bonded acids coordinated to the cluster.[S1] Note that some ligands are removed for clarity.

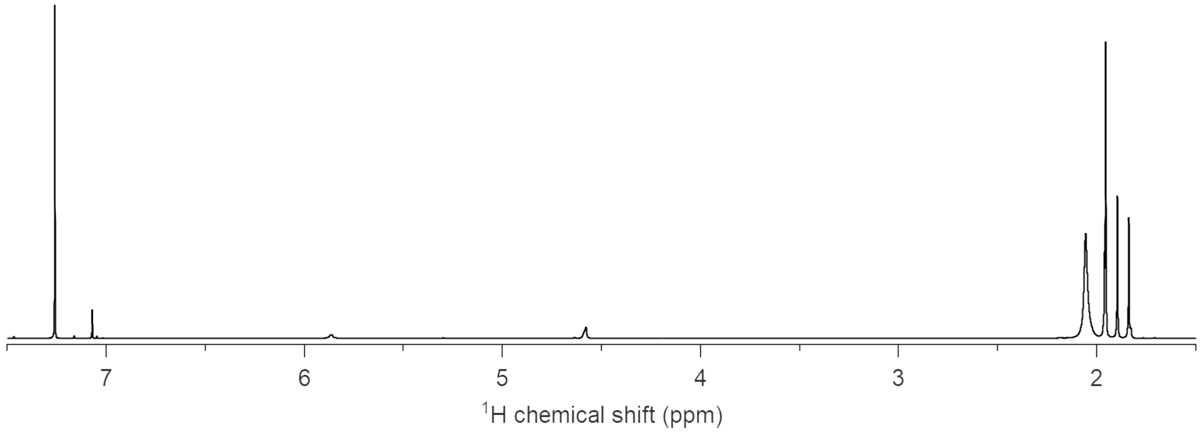

Figure S10: ^{1}H-NMR spectrum of the **Zr12**-acetate cluster. There is no observable signal at 4 ppm indicating that the amount of ester impurity is very low.

In an effort to remove all the hydrogen bonded (protonated) acid from a **Zr12**-oleate cluster a non-nucleophilic base (1,8-Diazabicyclo[5.4.0]undec-7-ene or DBU) was added. The base can accept a proton from the hydrogen bonded oleic acid and thus detach it from the cluster. Afterwards, size exclusion chromatography (SEC) was performed to separate the clusters from the DBU coordinated acid. Only the first fraction of the SEC was obtained as a pure

compound (absence of the peak around 1700 cm^{-1}) with a very low yield of 0.8%. From the second fraction onwards, H-bonded acid can be seen in the spectra, see figure S11).

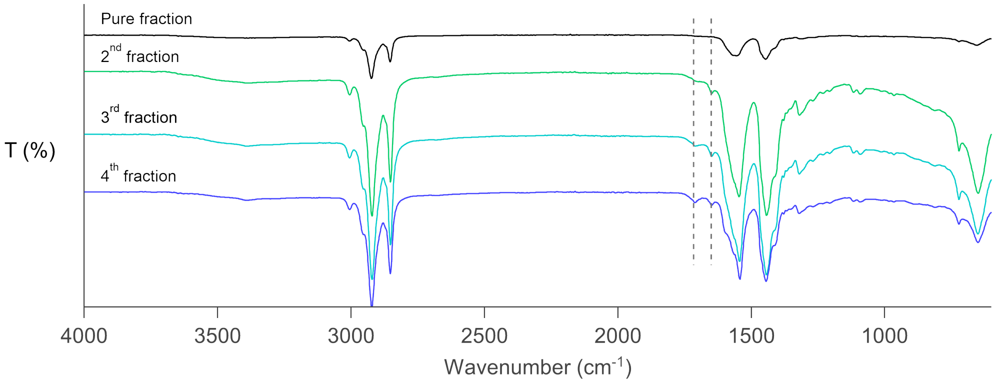

Figure S11: Fractions collected with size exclusion chromatography

To quantify the amount of hydrogen bonded acid, we measured TGA of our synthesized clusters, see Figure S12.

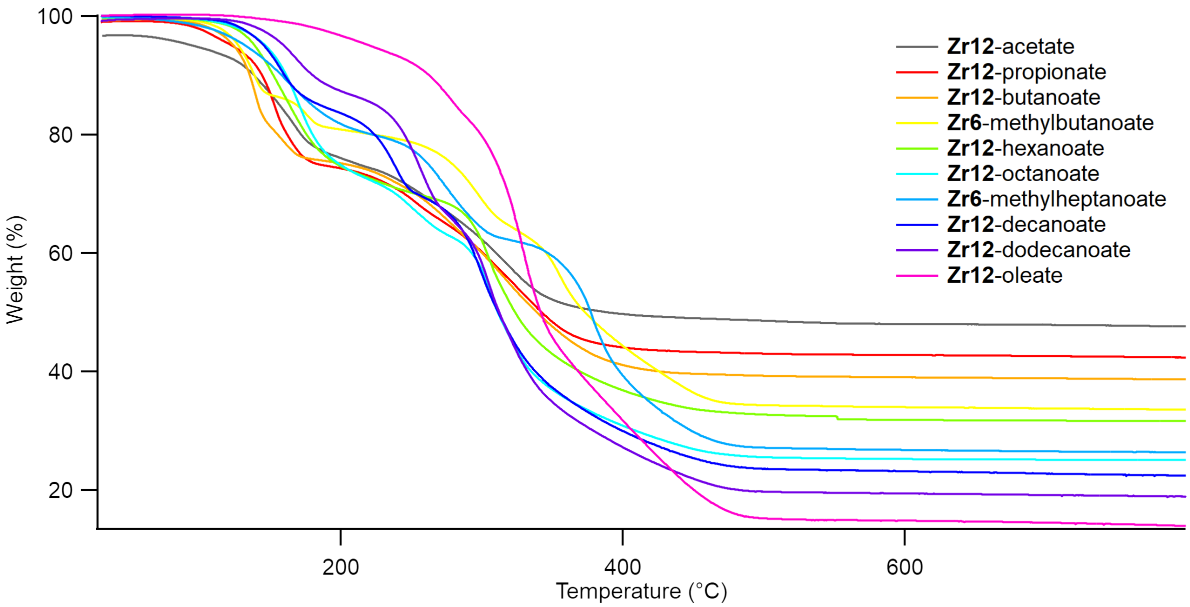

Figure S12: Thermogravimetric analysis of our bottom up synthesized zirconium oxo clusters.

Assuming a pure cluster, the theoretical mass loss can be calculated from the molecular

formula, considering that the end product is zirconia.

$$Zr_6O_8H_4(OOCR)_{12} \longrightarrow 6ZrO_2 + \text{byproducts} \tag{1}$$

Starting from 100 g of clusters, we can calculate the mass of zirconia at the end:

$$m_{\mathrm{ZrO_2}} = (\frac{100}{M_{cluster}}) \times 6 \times M_{\mathrm{ZrO_2}} \tag{2}$$

Where $M_{cluster}$ and $M_{\mathrm{ZrO_2}}$ are the molecular weights of the cluster and zirconia, respectively. Note that the calculations are done with the monomeric species and we use the molecular weight of the monomer (which is exactly half of the dimer) for all our calculations. This value is reported in Table S7 as the theoretical value. The experimental value is consistently lower than the theoretical one (Table S7), indicating an extra organic fraction that is assigned to mostly hydrogen bonded ligands. We quantify its amount by the following procedure. Again assuming that we start from 100 g of clusters, we calculated the molar amount of zirconia in the residual mass (experimental value).

$$n_{\mathrm{ZrO_2}} = \frac{mass}{M_{\mathrm{ZrO_2}}} \tag{3}$$

We determine the molar amount of monomeric cluster that this corresponds to:

$$n_{cluster} = \frac{n_{\mathrm{ZrO_2}}}{6} \tag{4}$$

We calculate the apparent molecular weight of the cluster by using the molar amount and the starting mass (100 g):

$$M_{apparent} = \frac{100g}{n_{cluster}} \tag{5}$$

The difference with the theoretical molecular weight is calculated and assigned to the extra

organic fraction:

$$\Delta M = M_{apparent} - M_{theoretical} \tag{6}$$

By dividing ΔM by the molecular weight of the carboxylic acid, we get the number of carboxylic acids that is present per monomer.

$$\frac{\text{extra acid}}{monomer} = \frac{\Delta M}{M_{acid}} \tag{7}$$

This final value is also reported in Table S7.

Table S7: TGA data on bottom up synthesized clusters. The values in the table is the remaining mass of ZrO_2 in %. Note that for the acetate cluster the value in the table is on top of the extra acid from the crystal structure $Zr_{12}O_8(OH)_8(CH_3COO)_{24}.6\,CH_3COOH.3.5\,DCM$.

Ligand	Theoretical value (%)	experimental value (%)	Extra acid/monomer
Acetic acid	53.3	48	2.5
Propionic acid	47.5	42.8	2.3
Butyric acid	42.9	39	1.9
Methylbutyric acid	39	34	2.8
Hexanoic acid	35.9	31.9	2.2
Octanoic acid	30.8	25.2	3.7
Methyl heptanoic acid	30.8	29.1	1.1
Decanoic acid	27	23.2	2.6
Dodecanoic acid	24.1	19.4	3.7
Oleic acid	18.2	14.8	3.4

4 NMR analysis

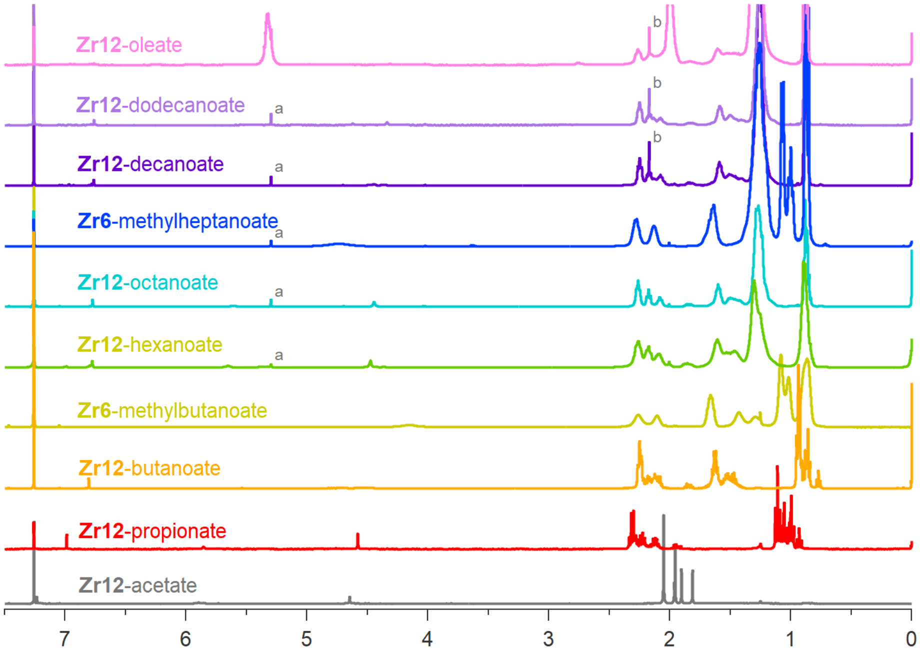

Figure S13: ^{1}H NMR spectra in $CDCl_3$ of **Zr12**-butanoate, octanoate, decanoate, dodecanoate and **Zr6**-methylbutanoate. The peaks indicated with a is remaining DCM, the peaks indicated with b is remaining acetone.

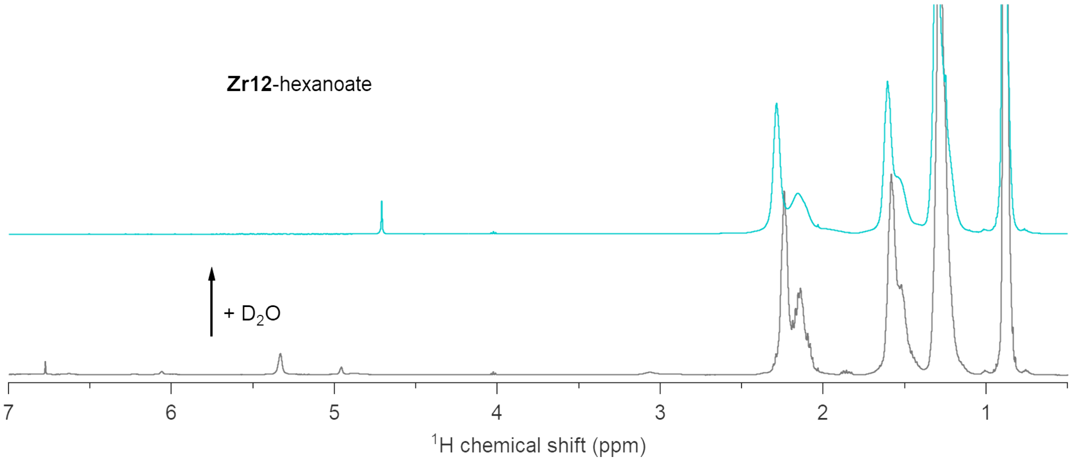

Figure S14: we added a small amount of D_2O (20 µL) to a solution of **Zr12**-hexanoate cluster in $CDCl_3$. Upon addition of D_2O, the deuterium rapidly exchanges for the protons and all the signals collapse onto a single resonance at 4.77 ppm

Table S8: Determination of the contribution of homogeneous and heterogeneous broadening for three different resonances in the **Zr12**-oleate cluster. We report here the experimental FWHM, the experimentally determined T_2 relaxation time contanst, the calculated homogeneous line width and finally the homogeneous broadening of the multiplet resonances after simulation of the spectrum with the calculated homogeneous line width. We find that the homogeneous broadening is the main contributor and there is little heterogeneous broadening present.

Resonance	FWHM (Hz)	T_2 (ms)	$1/(\pi T_2)$ (Hz)	$\Delta\nu$
αCH_2	25.0	30.57	8.92	20
Alkene	11.1	200.0	1.59	9
Methyl	1.53	590.2	0.54	2

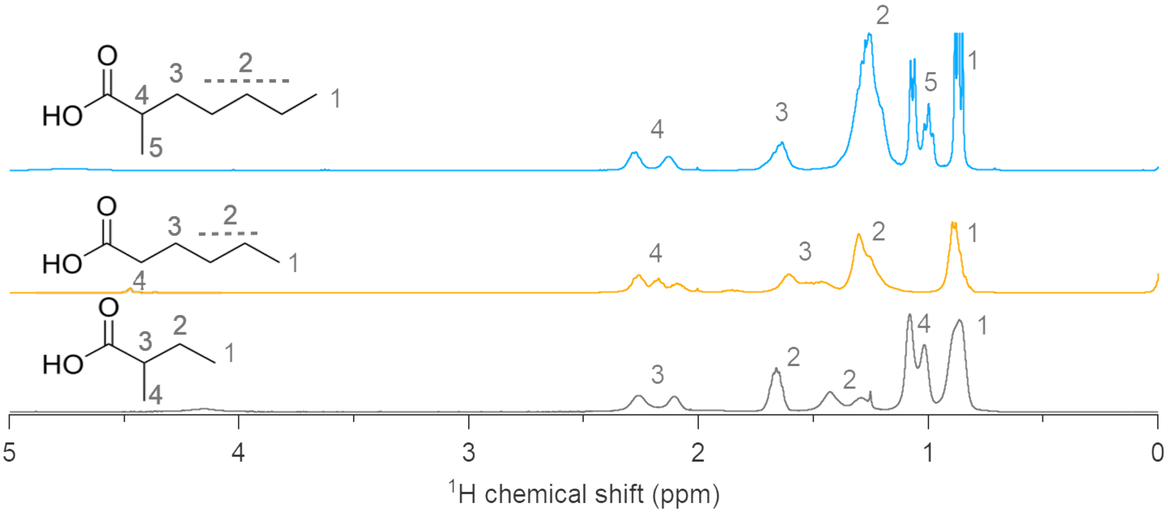

Figure S15: ^{1}H NMR of **Zr6**-methylbutanoate, -methylheptanoate and **Zr12**-hexanoate with assigned peaks.

5 HR-MS

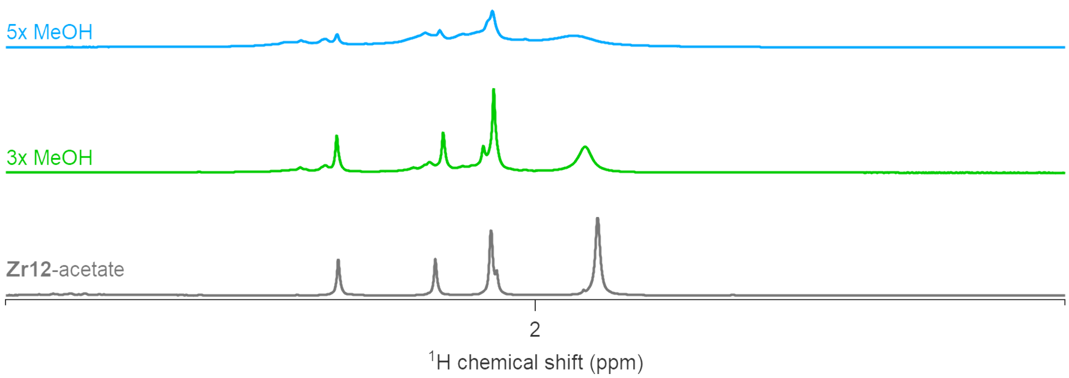

Figure S16: ^{1}H NMR of the **Zr12**-acetate cluster in CD_3OD. All signals disappear after multiple cycles of dissolving/evaporation indicating degradation of the cluster.

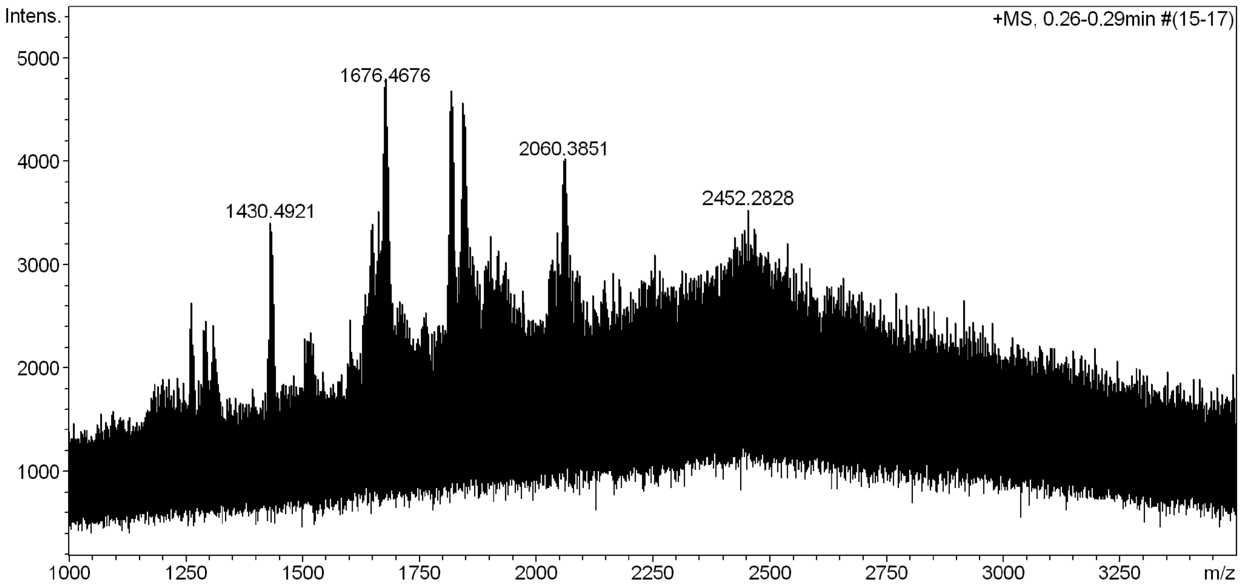

Figure S17: HRMS of the **Zr12**-acetate cluster in MeOH, no signals are close to the target mass (2775.17 g/mol) or could be matched with degradation products.

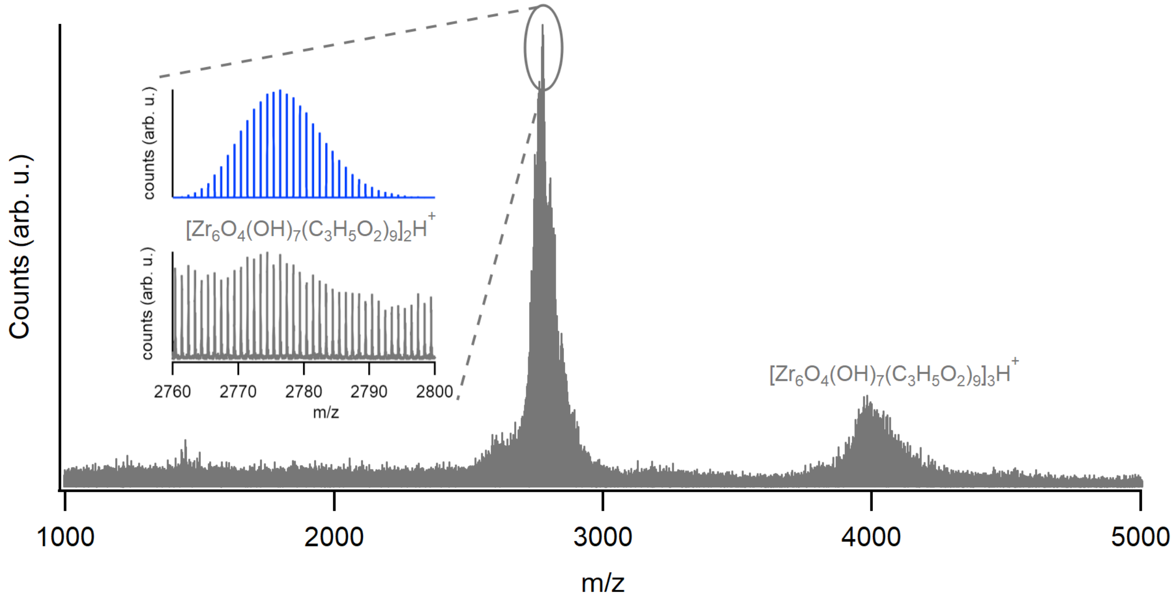

Figure S18: HRMS of the bottom up synthesized **Zr12**-propionate cluster in THF, the grey spectrum (experimental) is compared with the blue spectrum (simulated).

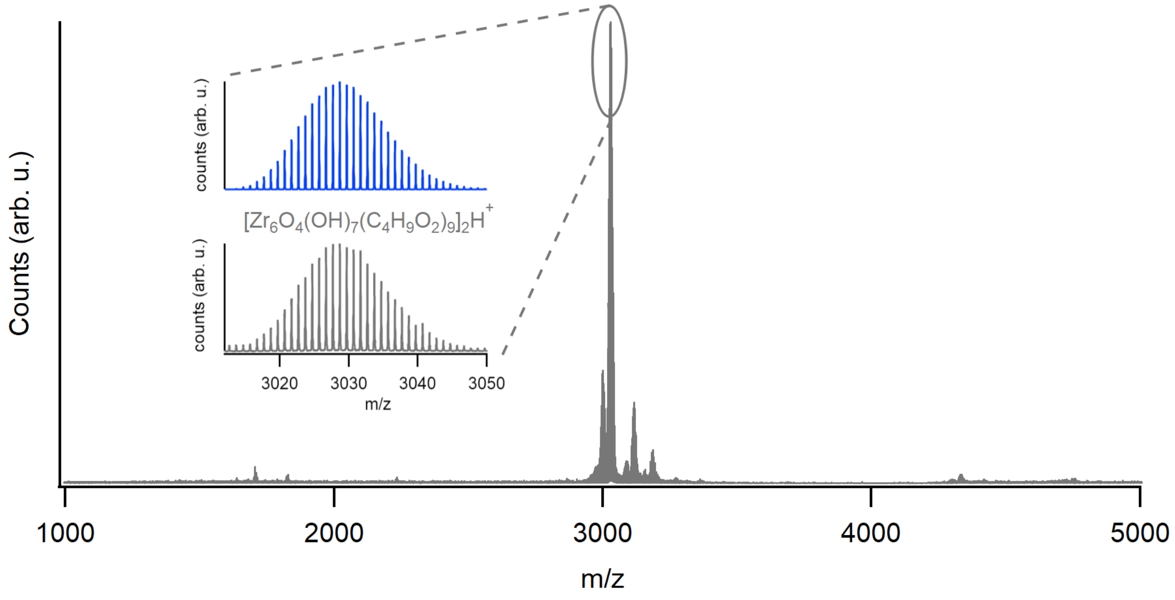

Figure S19: HRMS of the bottom up synthesized **Zr12**-butanoate cluster in THF, the grey spectrum (experimental) is compared with the blue spectrum (simulated).

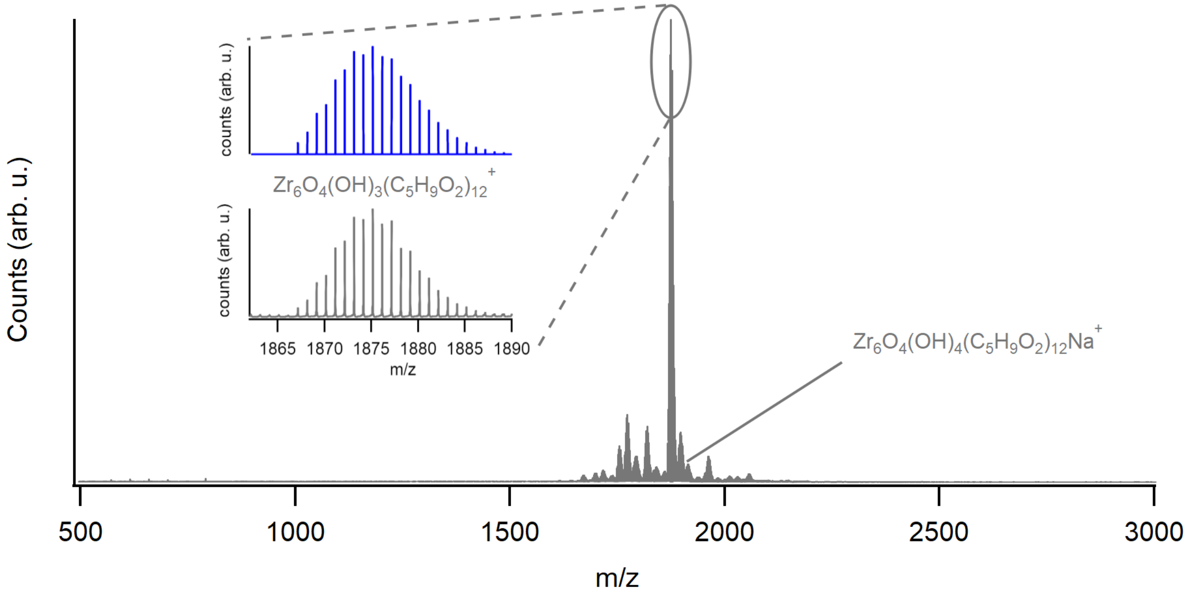

Figure S20: HRMS of the bottom up synthesized **Zr6**-methylbutanoate cluster in THF, the grey spectrum (experimental) is compared with the blue spectrum (simulated).

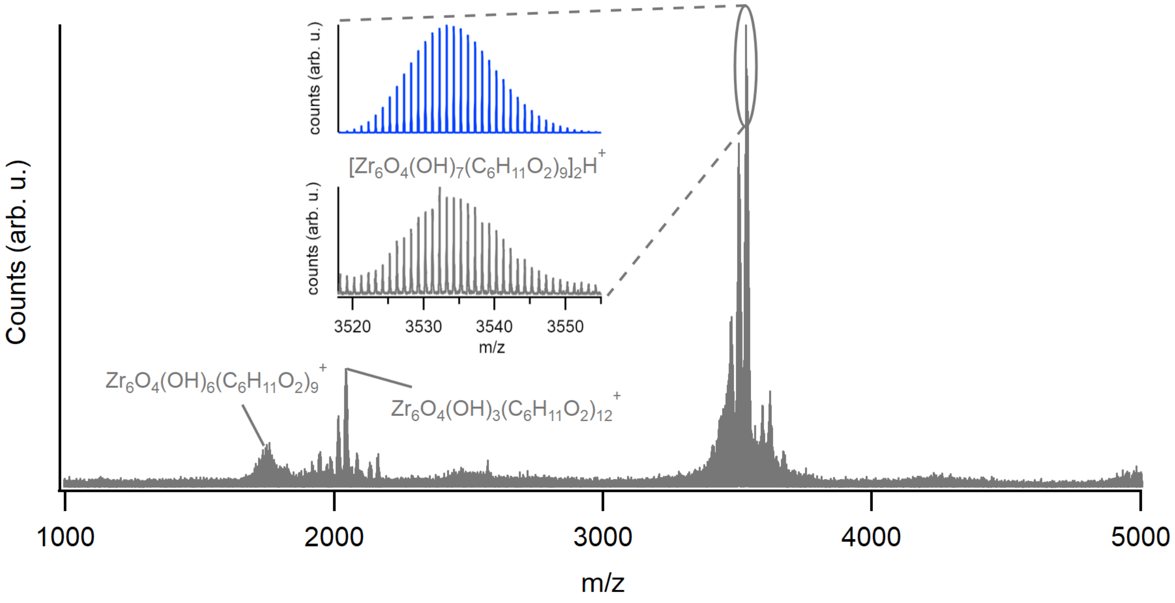

Figure S21: HRMS of the bottom up synthesized **Zr12**-hexanoate cluster in THF, the grey spectrum (experimental) is compared with the blue spectrum (simulated).

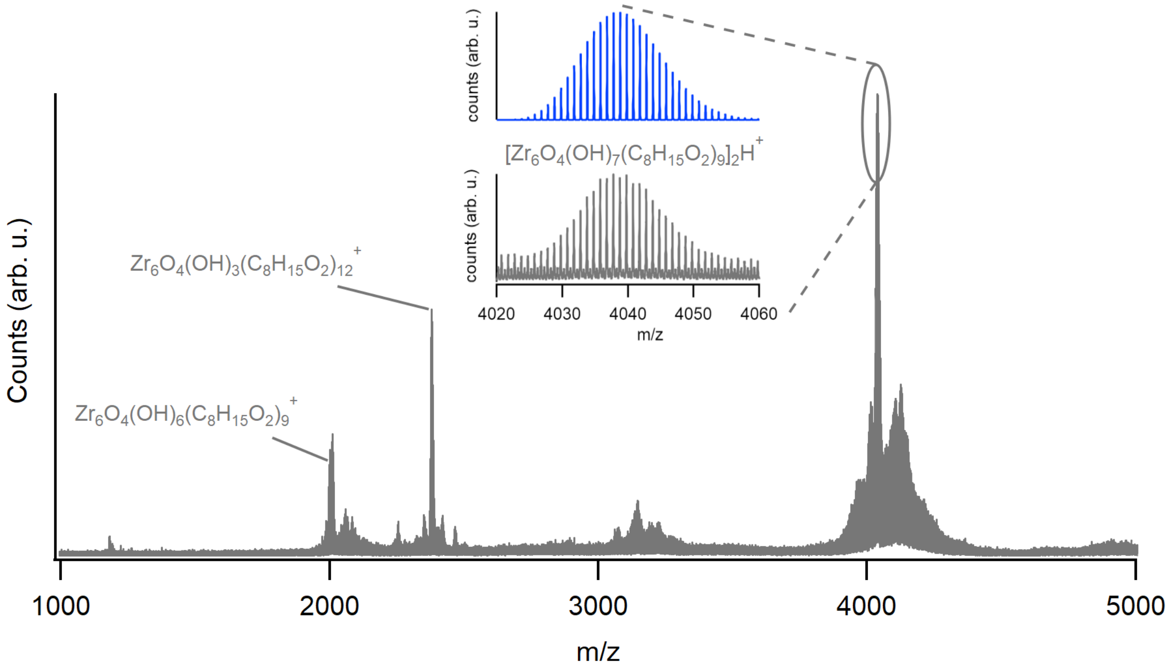

Figure S22: HRMS of the bottom up synthesized **Zr12**-octanoate cluster in THF, the grey spectrum (experimental) is compared with the blue spectrum (simulated).

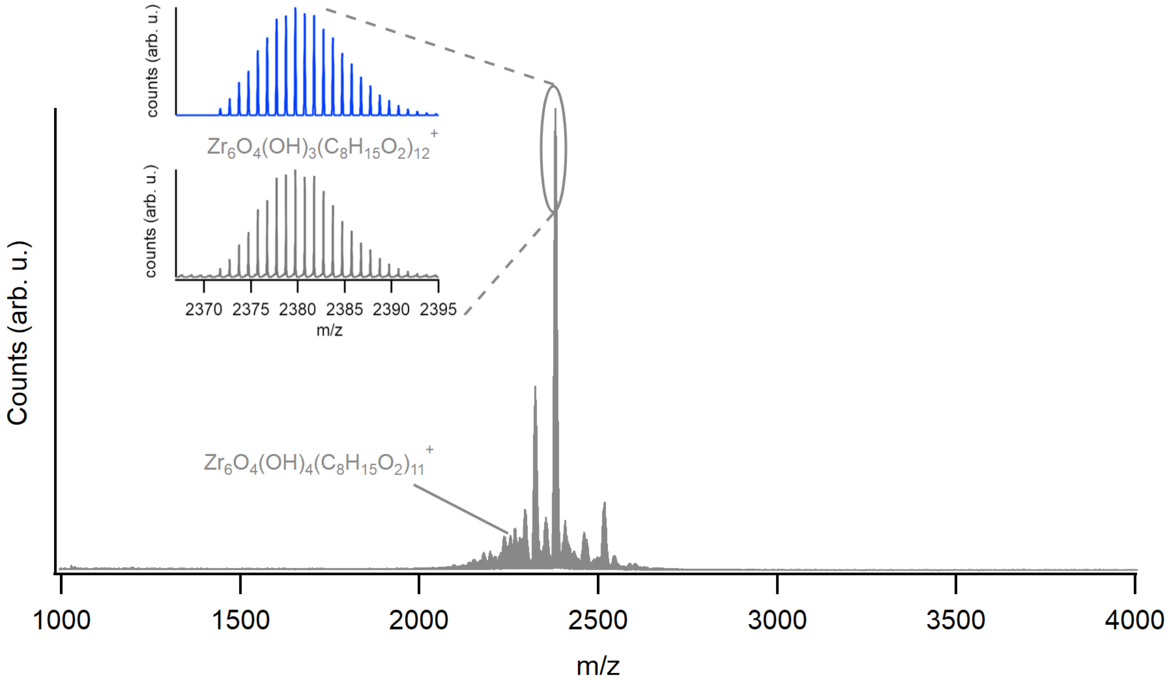

Figure S23: HRMS of the bottom up synthesized **Zr6**-methylheptanoate cluster in THF, the grey spectrum (experimental) is compared with the blue spectrum (simulated).

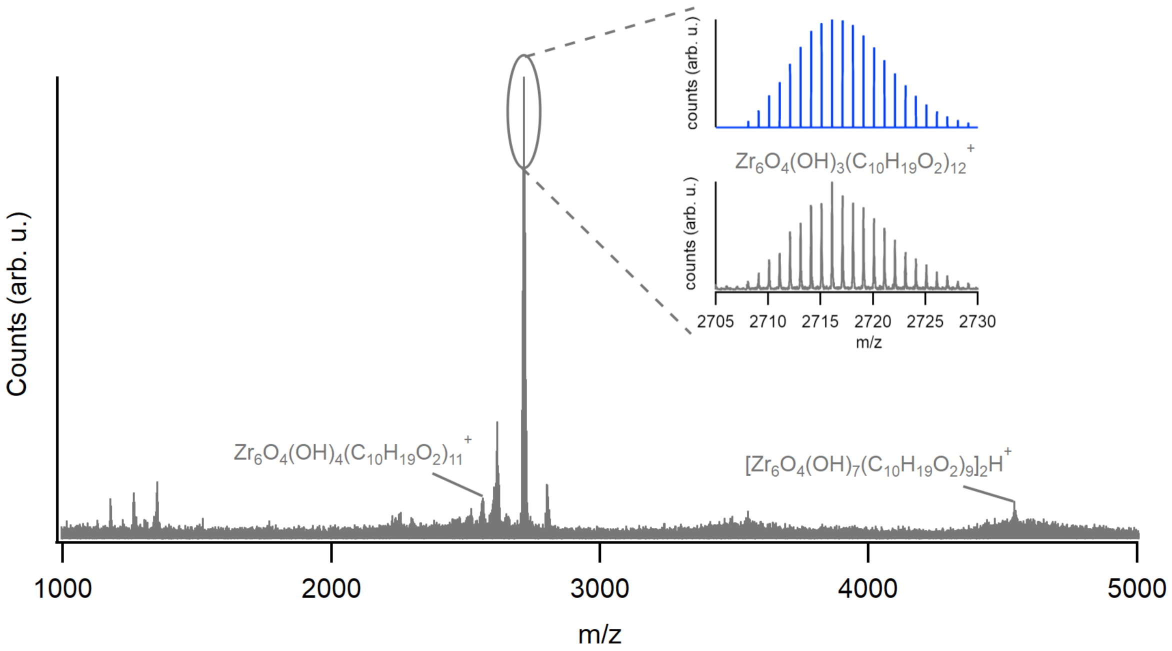

Figure S24: HRMS of the bottom up synthesized **Zr12**-decanoate cluster in THF, the grey spectrum (experimental) is compared with the blue spectrum (simulated).

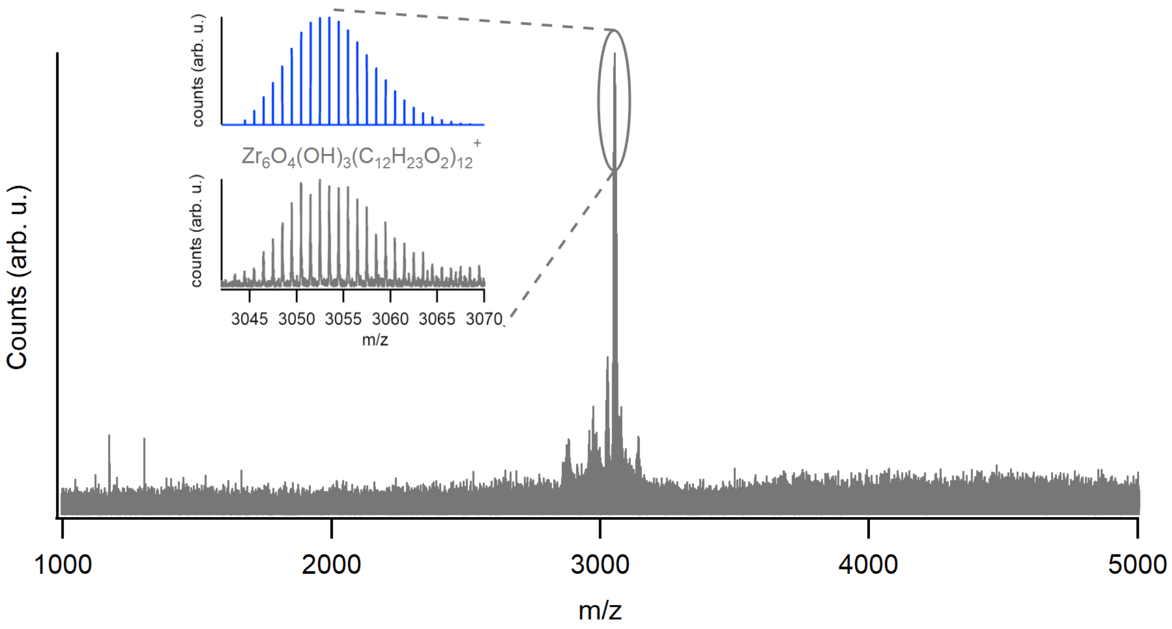

Figure S25: HRMS of the bottom up synthesized **Zr12**-dodecanoate cluster in THF, the grey spectrum (experimental) is compared with the blue spectrum (simulated).

6 Clusters synthesized by ligand exchange

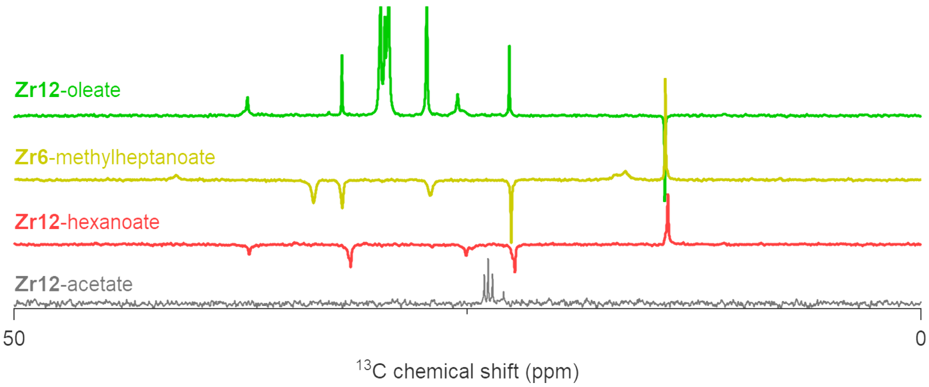

Figure S26: Overlay C-NMR for the **Zr12**-hexanoate, -oleate and **Zr6**-methylheptanoate synthesized via exchange reaction. **Zr12**-acetate is added as a reference to prove that the signals of acetate are gone after exchange.

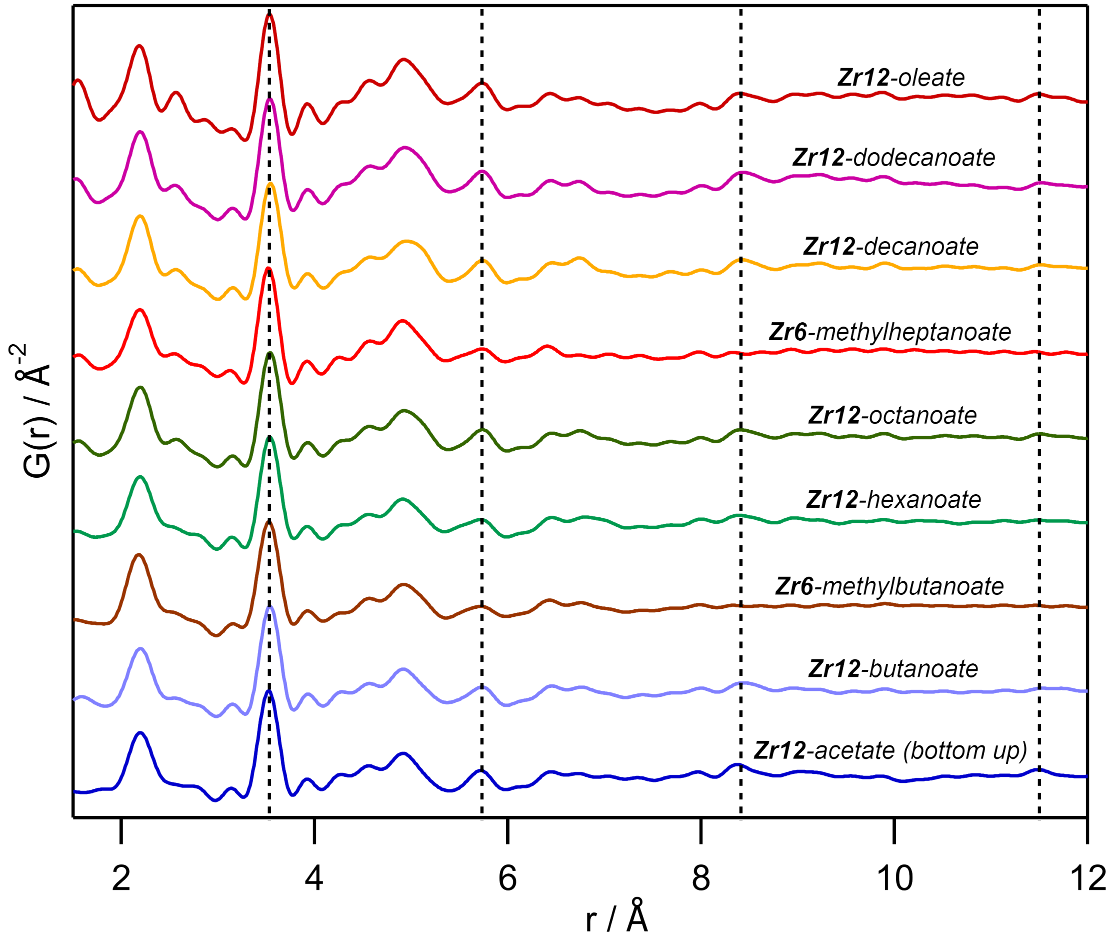

Figure S27: PDF spectra of the clusters synthesized via the exchange reaction. Note that the acetic acid spectrum is added as a reference spectrum as it is not synthesized via an exchange reaction.

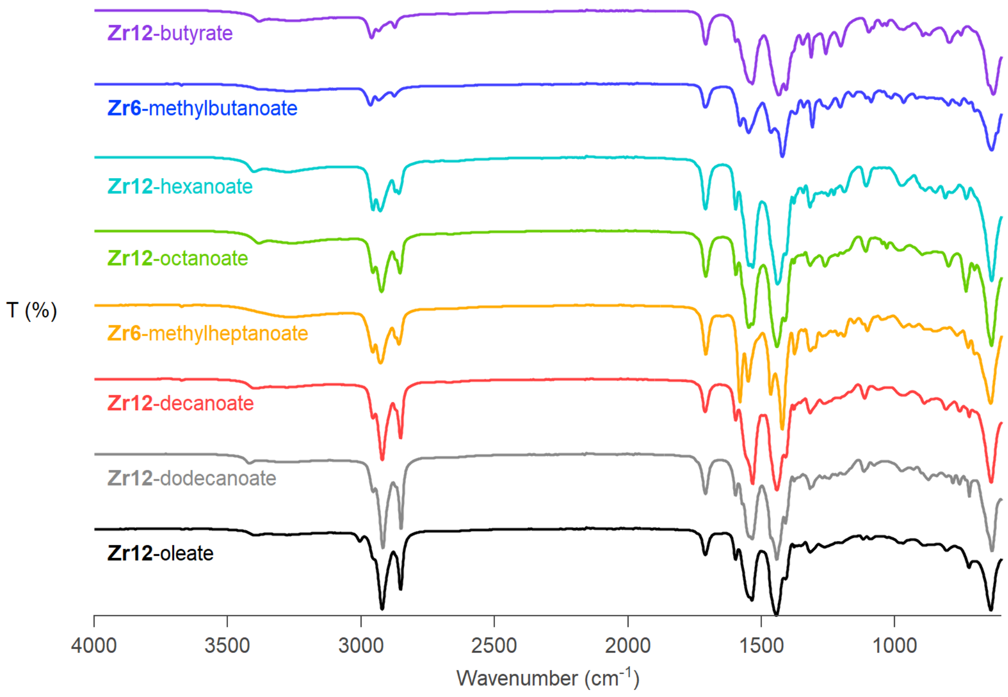

Figure S28: IR spectra of the clusters synthesized via the exchange reaction.

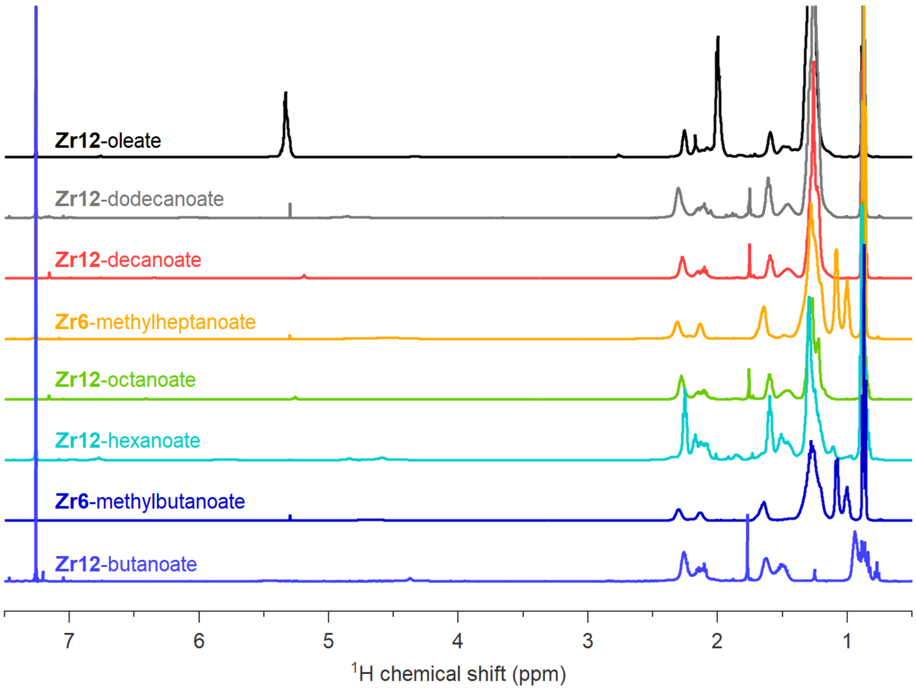

Figure S29: NMR spectra of the clusters synthesized via the exchange reaction.

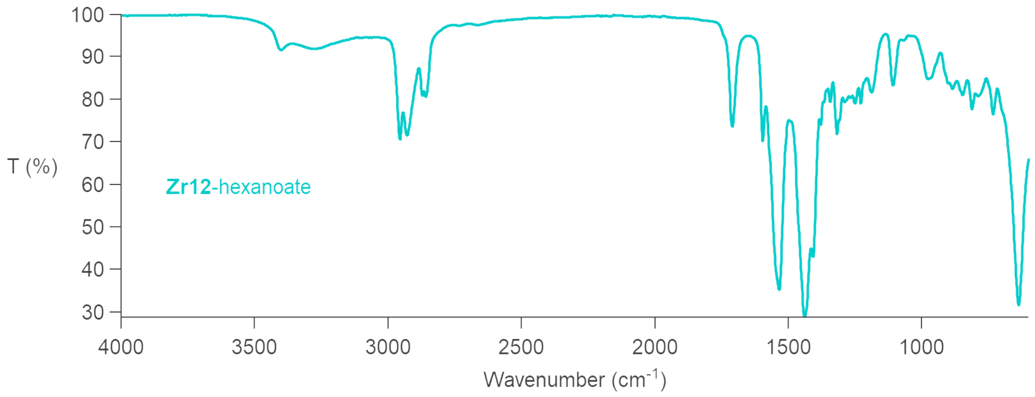

Figure S30: FTIR spectrum of the cluster obtained via exchanging **Zr6**-methylbutanoate with hexanoic acid.

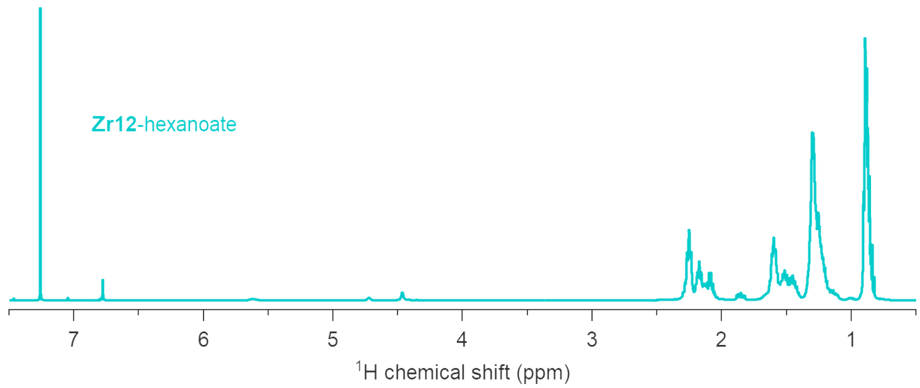

Figure S31: NMR spectrum of the cluster obtained via exchanging **Zr6**-methylbutanoate with hexanoic acid.

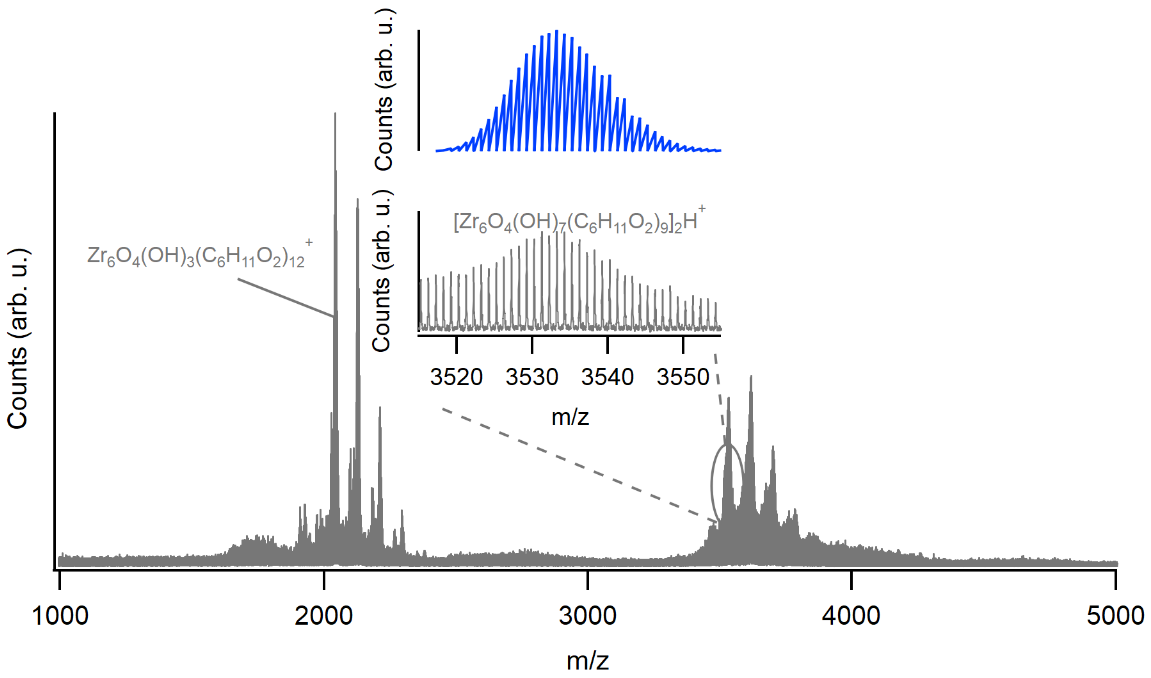

Figure S32: MS spectrum of the cluster obtained via exchanging **Zr6**-methylbutanoate with hexanoic acid.

7 Hafnium oxo clusters

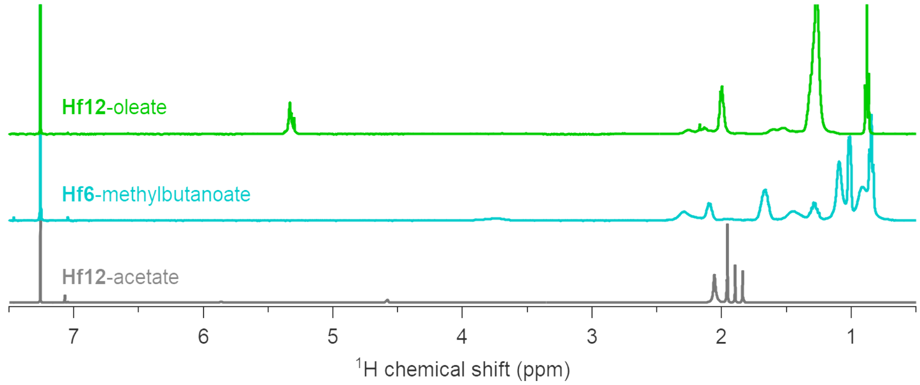

Figure S33: Overlay NMR for the **Hf12**-acetate, **Hf6**-methylbutanoate and the **Hf12**-oleate obtained via a bottom up reaction.

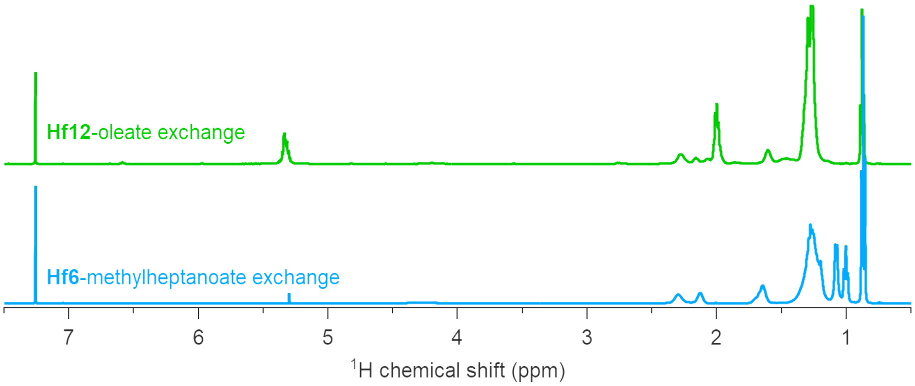

Figure S34: Overlay NMR for the **Hf6**-methylheptanoate and the **Hf12**-oleate obtained via an exchange reaction from **Hf12**-acetate.

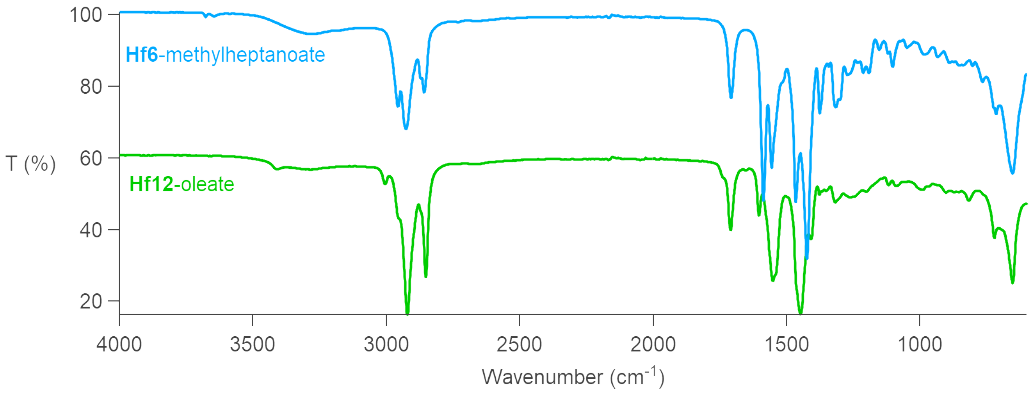

Figure S35: Overlay IR for the **Hf6**-methylheptanoate and the **Hf12**-oleate obtained via an exchange reaction from **Hf12**-acetate.

Table S9: Refined parameters after fitting hafnium cluster with different ligand cappings with **Hf12/Hf6**-acetate model. **Hf6**-acetate model was created by removing atoms from **Hf12**-acetate model.

	**Hf12**-acetate	**Hf6**-methylbutanoate	**Hf12**-oleate
**Model**	**Hf12**-acetate	**Hf6**-acetate	**Hf12**-acetate
**Scale**	1.23	1.33	1.24
**Uiso Hf (Å^2)**	0.005	0.005	0.006
**Uiso O (Å^2)**	0.016	0.028	0.024
**Uiso C (Å^2)**	0.015	0.020	0.012
**delta2 (Å^2)**	3.11	3.44	3.68
**Rw**	0.10	0.10	0.12
**Amplitude (A)**		2.314	1.251
**wasyn**		0.407	-0.145
λ		3.469	3.974
ϕ		0.268	-4.891
θ		4.736	5.120
**wsig**		0.240	-0.532

Table S10: Bond distances in acetate capped hafnium and zirconium oxo clusters. M=Zr/Hf.[S1]

	**Zr12**-acetate	**Hf12**-acetate
M-O	2.033-2.396	2.031-2.405
M-M Intra 1	3.448-3.588	3.448-3.588
M-M Intra 2	4.953-4.969	4.953-4.969
M-M Inter 1	5.588-5.684	5.588-5.684
M-M Inter 2	8.270-8.609	8.270-8.609
M-M Inter 3	11.256-11.956	11.256-11.956

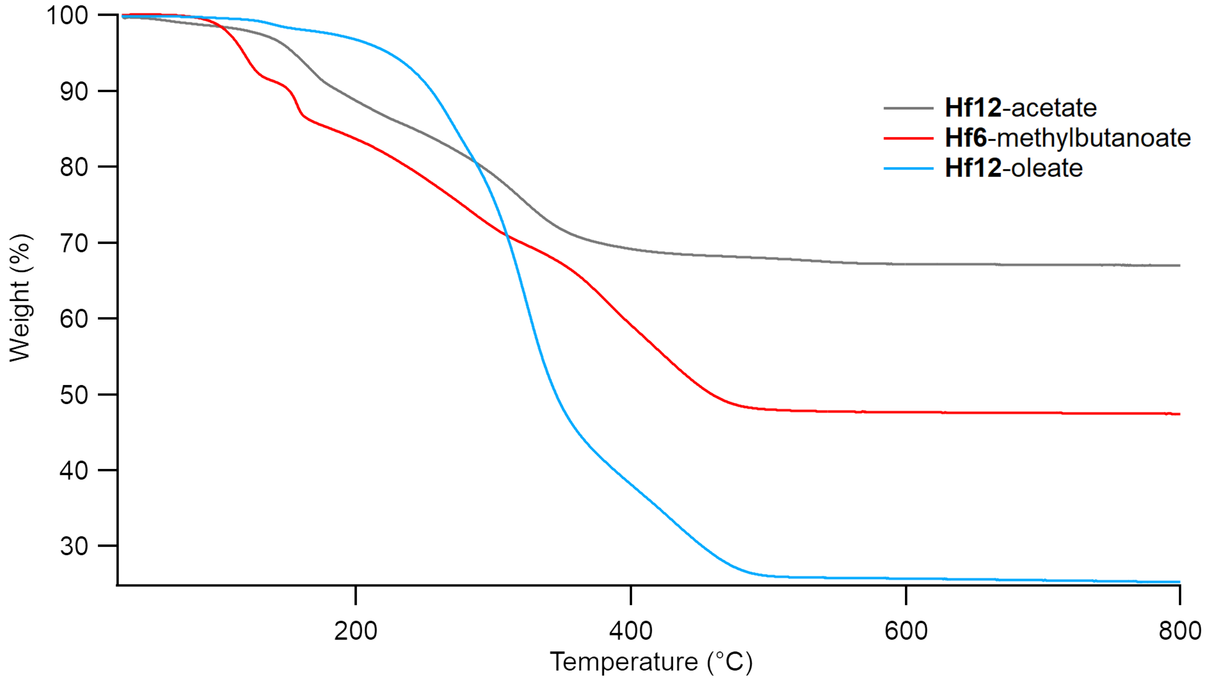

Figure S36: Thermogravimetric analysis of our bottom up synthesized hafnium clusters.

Table S11: TGA data on bottom up synthesized Hafnium clusters. The values in the table is the remaining mass of HfO_2 in % and are calculated in the same way as shown before for ZrO_2 by replacing $M_{cluster}$ and $M_{\mathrm{ZrO_2}}$ with its hafnium counterpart.

Ligand	Theoretical value (%)	experimental value (%)	Excess acid/monomer
Acetic acid	66.1	65.7	0.2
Methylbutyric acid	52.3	47.7	2.3
Oleic acid	27.6	25.7	1.2

8 Zirconium oxide nanocrystals

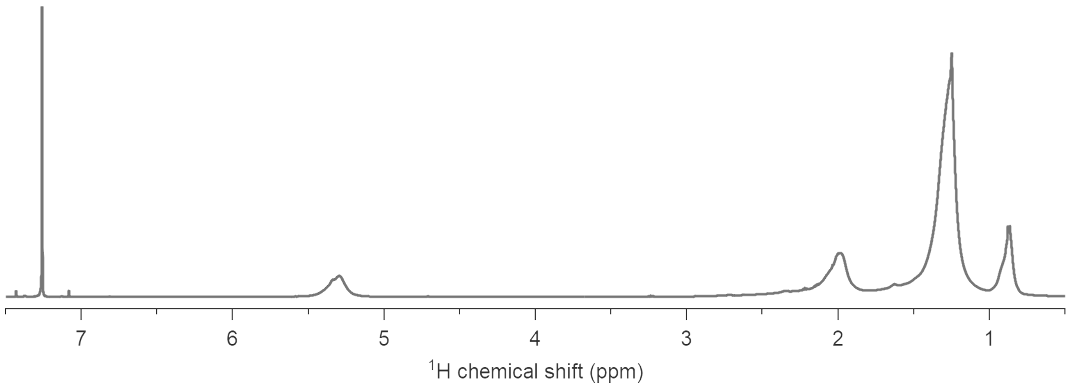

Figure S37: ^{1}H NMR spectrum of ZrO_2 nanocrystals capped with oleic acid.

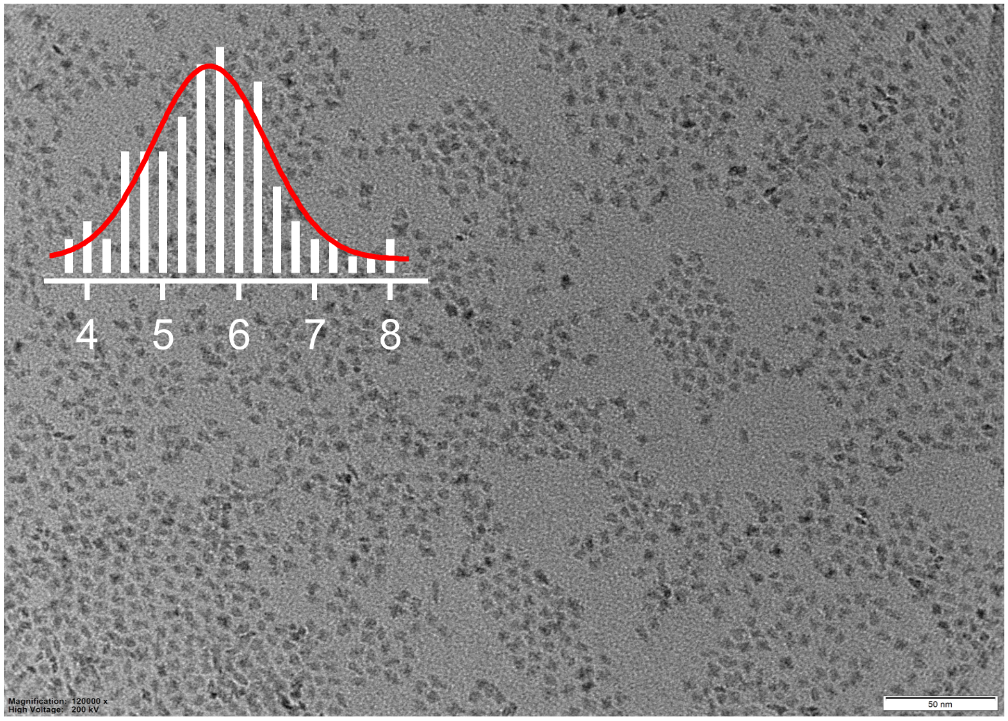

Figure S38: TEM image of ZrO_2 nanocrystals capped with oleic acid, with the histogram as inset.

References

(S1) Puchberger, M.; Kogler, F. R.; Jupa, M.; Gross, S.; Fric, H.; Kickelbick, G.; Schubert, U. Can the Clusters Zr6O4(OH)4(OOCR)12 and [Zr6O4(OH)4(OOCR)12]2 Be Converted into Each Other? *European Journal of Inorganic Chemistry* **2006**, *2006*, 3283–3293.
